# Core transcription regulatory circuitry orchestrates corneal epithelial homeostasis

**DOI:** 10.1038/s41467-020-20713-z

**Published:** 2021-01-18

**Authors:** Mingsen Li, Huaxing Huang, Lingyu Li, Chenxi He, Liqiong Zhu, Huizhen Guo, Li Wang, Jiafeng Liu, Siqi Wu, Jingxin Liu, Tao Xu, Zhen Mao, Nan Cao, Kang Zhang, Fei Lan, Junjun Ding, Jin Yuan, Yizhi Liu, Hong Ouyang

**Affiliations:** 1 State Key Laboratory of Ophthalmology, Zhongshan Ophthalmic Center, Sun Yat-sen University, 510060 Guangzhou, China; 2 Key Laboratory of Epigenetics and Metabolism, Ministry of Science and Technology, Institutes of Biomedical Sciences; Liver Cancer Institute, Zhongshan Hospital, Fudan University, 200032 Shanghai, China; 3 Center for Stem Cell Biology and Tissue Engineering, Key Laboratory for Stem Cells and Tissue Engineering, Ministry of Education, Sun Yat-Sen University, 510080 Guangzhou, China; 4 Program of Stem Cells and Regenerative Medicine, Fifth Affiliated Hospital, Zhongshan School of Medicine, Sun Yat-Sen University, Guangdong, China; 5 Center for Biomedicine and Innovations, Faculty of Medicine, Macau University of Science and Technology, Macau, China; 6 Research Units of Ocular Development and Regeneration, Chinese Academy of Medical Sciences, Beijing, China

**Keywords:** Epigenetics, Transcription, Transcriptomics, Adult stem cells, Self-renewal

## Abstract

Adult stem cell identity, plasticity, and homeostasis are precisely orchestrated by lineage-restricted epigenetic and transcriptional regulatory networks. Here, by integrating super-enhancer and chromatin accessibility landscapes, we delineate core transcription regulatory circuitries (CRCs) of limbal stem/progenitor cells (LSCs) and find that RUNX1 and SMAD3 are required for maintenance of corneal epithelial identity and homeostasis. *RUNX1* or *SMAD3* depletion inhibits *PAX6* and induces LSCs to differentiate into epidermal-like epithelial cells. RUNX1, PAX6, and SMAD3 (RPS) interact with each other and synergistically establish a CRC to govern the lineage-specific *cis*-regulatory atlas. Moreover, RUNX1 shapes LSC chromatin architecture via modulating H3K27ac deposition. Disturbance of RPS cooperation results in cell identity switching and dysfunction of the corneal epithelium, which is strongly linked to various human corneal diseases. Our work highlights CRC TF cooperativity for establishment of stem cell identity and lineage commitment, and provides comprehensive regulatory principles for human stratified epithelial homeostasis and pathogenesis.

## Abstract (web summary)

Corneal epithelium shares similar molecular signatures to other stratified epithelia. Here, the authors map super-enhancers and accessible chromatin in corneal epithelium, identifying a transcription regulatory circuit, including RUNX1, PAX6, and SMAD3, required for corneal epithelial identity and homeostasis.

## Introduction

Mammalian stratified squamous epithelia, consisting of multiple layers of epithelial cells, line the external surfaces of various organs. The integrity and function of these epithelial tissues are maintained by stem cells residing in the basal layer^1^. Stem cell identity and fate commitment are dominated by lineage-restricted transcription factor (TF) network and specific epigenetic architecture^2–4^. In a given cell type, the core TFs form an interconnected autoregulatory loop termed core transcription regulatory circuitry (CRC), in which the TFs self-regulate via binding to their own super-enhancers (SEs) and the TFs themselves bind to the SEs of one another^5^. Recent studies have suggested that CRC model plays a key role in tissue development, homeostasis, and disease progression^5–7^. To date, the mechanism that CRC TF cooperativity regulates stratified epithelial stem cell identity and specification is largely unknown.

The corneal epithelium consists of non-keratinized stratified squamous epithelial cells and shares many similarities in function, maintenance of homeostasis, and molecular signature with other stratified epithelia^8^. Limbal stem/progenitor cells (LSCs) segregated in the basal layer of limbus undergo self-renewal and differentiation throughout life to maintain corneal epithelial homeostasis and regeneration^9,10^, which is orchestrated by lineage-specific regulators^11–13^. TP63, a master stratified epithelial regulator, initiates stratification program and maintains the self-renewal capacity of stratified epithelial stem cells^14–16^. The corneal epithelial-specific master regulator PAX6 plays an indispensable role in the non-keratinizing characterization of LSCs^12,17^. Dysfunction of LSCs caused by various insults, such as inflammation and chemical injury, accompanied by replacement of opacified keratinized epithelium and neovascularization, leads to severe visual impairment and blindness in millions of individuals worldwide^8,17^.

Here, using the corneal epithelium as a model tissue, we characterize the SE-mediated regulatory landscape of LSCs and reveal a CRC TF interconnected auto-regulation network. We then show that RUNX1, PAX6, and SMAD3 (RPS) proteins interact physically and establish an enhancer especially SE repertoire, conferring corneal epithelial function and identity. RUNX1 also directs histone acetylation at enhancers to drive transcription of LSC-specific genes. Dysfunction of RPS cooperation results in cell fate switching from the corneal epithelium to an epidermal type. Altogether, our work highlights the CRC TF-mediated regulatory networks for stratified epithelial lineage commitment and homeostasis.

## Results

### Chromatin regulatory landscape underlies corneal epithelial identity and homeostasis

**Fig. 1:**
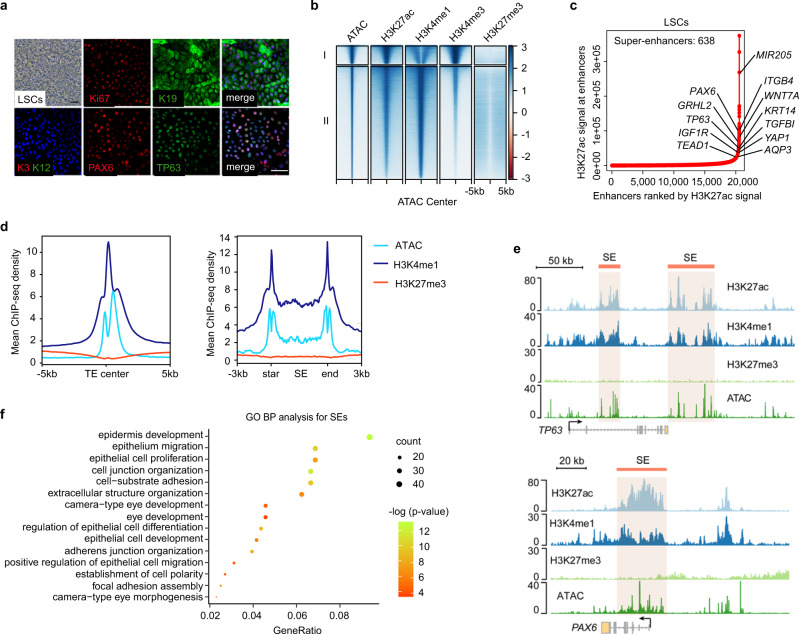
Histone modification and chromatin accessibility landscapes of primary LSCs. **a** Phase contrast image and immunofluorescence staining of primary LSCs for the indicated marker genes. Scale bar, 100 μm. **b** Heatmaps grouped into two clusters by *k*-means algorithm for ATAC-seq and the indicated ChIP-seq signals at the centers of ATAC peaks in LSCs. **c** Ranked enhancer plots defined by H3K27ac. Enhancers above the inflection point of the curve have exceptionally strong H3K27ac signals and are defined as SEs. The selected genes are SE-associated genes. **d** Metaplots of average ATAC-seq, H3K4me1, and H3K27me3 density at TEs (left) and SEs (right) in LSCs. **e** Genome browser tracks for H3K27ac, H3K4me1, H3K27me3, and ATAC-seq signals at the indicated SE loci. **f** GO biological process (BP) analysis of SE-associated genes with pvalueCutoff = 0.01 and qvalueCutoff = 0.05.

To map the chromatin landscape of LSCs, we used a defined feeder-free culture system to generate homogeneous primary LSCs with robust expression of the markers PAX6, TP63, and KRT19 and absence in the differentiated corneal epithelial cell (CEC) markers KRT3 and KRT12^17^ (Fig. 1a). These cells proliferated rapidly in vitro, as evidenced by a large fraction of cells expressing the mitotic marker Ki67 (Fig. 1a).

We then employed chromatin immunoprecipitation sequencing (ChIP-seq) to dissect the histone modifications (H3K27ac, H3K4me1, H3K4me3, and H3K27me3) of LSCs, and also defined the accessible DNA elements by assay for transposase-accessible chromatin with sequencing (ATAC-seq)^18^. Unbiased hierarchical clustering revealed that ATAC hypersensitive sites (HSs) correlated highly with H3K27ac enrichment (Supplementary Fig. 1a). Similar to H3K27ac and H3K4me1 occupancy, the ATAC peaks were mainly located at intron and intergenic regions with a small fraction at proximal promoters, which was distinct from the H3K4me3 pattern showing the highest enrichment at promoters (Supplementary Fig. 1b, c). In contrast, most of the repressive H3K27me3 were enriched in the intergenic regions (Supplementary Fig. 1b). The accessible chromatin atlas was clustered into two groups: active promoters (high H3K27ac/H3K4me3 and low H3K4me1) and enhancers (high H3K27ac/H3K4me1 and low H3K4me3)^19^. Both clusters were mutually exclusive for H3K27me3 signal, indicative of active transcription events (Fig. 1b).

SEs are a category of closely stitched enhancer elements that are cell-type-specific and drive cell identity gene expression^20,21^. A set of large enhancer clusters with exceptionally high density of H3K27ac were defined as SEs, and the others were considered typical enhancers (TEs; Fig. 1c and Supplementary Fig. 1d). As expected, both SEs and TEs were enriched for high levels of ATAC and H3K4me1 signal, but lacked H3K27me3 mark (Fig. 1d, e), inferring high transcriptional activity. While TEs controlled the majority of LSC genes, SE-regulated genes were expressed at higher levels (Supplementary Fig. 1e). Gene ontology (GO) enrichment analysis showed that SEs of LSCs prominently governed genes linked to eye development and pan-epithelial biological processes, such as proliferation, migration, cell polarity, and cell junction organization (Fig. 1f). SE catalog was also enriched for the Hippo-YAP, TGFB, focal adhesion, and adherens junction signaling pathways (Supplementary Fig. 1f), which are critical to stratified epithelial function and homeostasis^22,23^. In addition to the distinguished stratified epithelial genes (*KRT14*, *ITGB4*, and *MIR-205*), we also cataloged the signaling molecules of WNT, TGFB, and NOTCH pathways in the SE gene set (Supplementary Fig. 1g, h), further highlighting their particular importance in stratified squamous epithelia^13,24^. Specifically, SE regulated *AQP3* and *IGF1R*, essential for corneal epithelial homeostasis^25,26^, and clinically well-recognized *TGFBI* responsible for human corneal structural integrity, transparency, and multiple corneal dystrophies^27,28^ (Fig. 1c).

Importantly, SEs drove a cohort of TFs including the well-established stratified epithelial regulators TP63 and GRHL2^29,30^ and key epidermal regulators KLF5^31^, AP-1 (JUNB/FOSL2)^32^, EHF^33^, and IRF6^34^ (Fig. 1e and Supplementary Fig. 1g), implying their potential preserved roles in stratified squamous epithelia. Of note, master TF *PAX6* also contained SE domain (Fig. 1e). Thus, SEs defined the identity, homeostasis, and lineage specification of LSCs. We speculated that additional core TFs critical for the corneal epithelium may be identified from the SE catalog.

### CRCs characterize interaction networks between stratification-related TFs and SEs

**Fig. 2:**
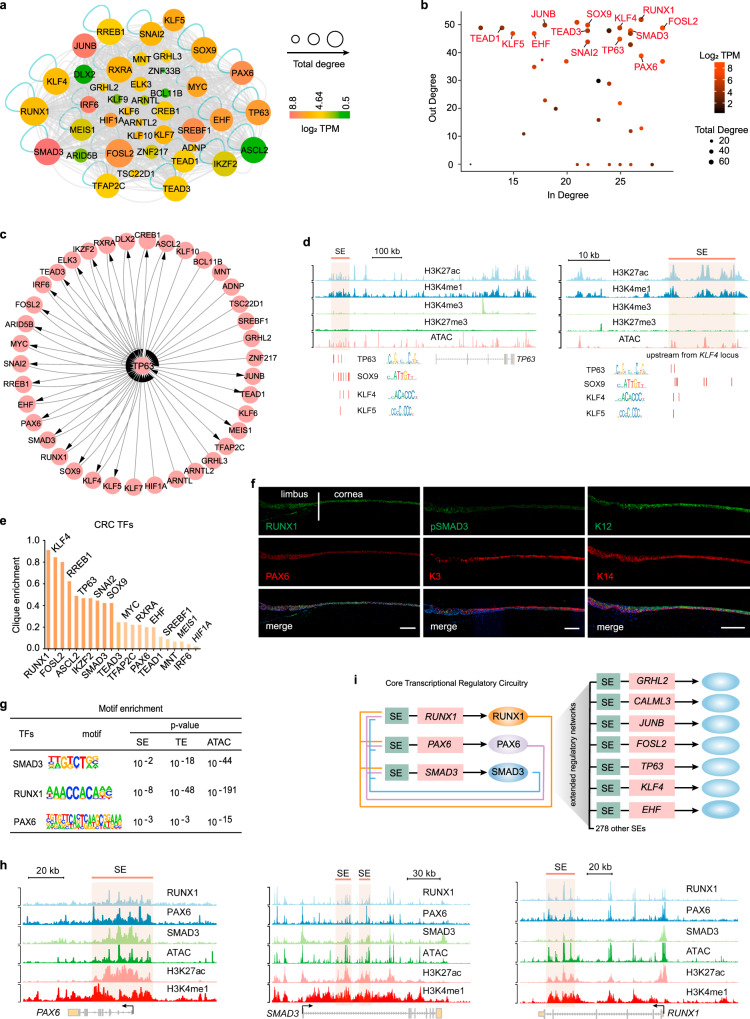
CRC model characterizes LSC-specific TF network. **a** Interaction network between TFs and SEs in LSCs. Each node is colored according to expression level (log_2_ TPM), and each node size is scaled according to total degree. Edges indicated in gray represent predicted interactions between TFs and SEs. The turquoise edges represent auto-regulatory loops. TPM: transcripts per kilobase million. **b** Scatter plot of in-degree (number of TFs binding to the SE of a node gene) and out-degree (number of SEs bound by a node TF) for genes in the SE-mediated regulatory network. **c** A TP63-centered network showing the SE-based regulatory relationships. **d** Genome browser tracks for the indicated histone modifications and ATAC signals at the indicated SE loci with logos and positions of the enriched TF motifs shown. **e** Clique enrichment score of each CRC TF calculated as the percentage of total cliques in which that TF is a constituent member. **f** Immunofluorescence staining of the indicated genes in normal adult human cornea and limbus. Scale bars, 200 μm. **g** Enrichment of RPS motifs at SEs, TEs, and ATAC peaks. **h** Genome browser tracks for the indicated ChIP-seq and ATAC-seq signals across the indicated SE loci in LSCs. **i** A CRC model established by RPS.

SEs and open chromatin regions are typically highly enriched for cell type-specific TFs^4,18,21^. The interactions of TFs with SEs or accessible DNA can define the interconnected TF networks that specify cell identity. Thus, we further reconstructed a SE-mediated transcription regulatory model in LSCs by integrating H3K27ac and ATAC data^6,7^ (Fig. 2a). The discrete accessible DNA elements within SEs were used to label TF-binding sites in our network analysis. The quantification of network connectivity between TF nodes embodied by in-degree (number of TFs binding to the SE of a node TF) and out-degree (number of SEs bound by a node TF) values revealed a set of highly connected TFs with robust expression, including PAX6, RUNX1, SMAD3, JUNB, FOSL2, TP63, KLF4, SOX9, EHF, TEAD1, and KLF5 (Fig. 2b). Most of them are also core regulators of other squamous epithelial tissues, implying the preserved function of this interaction network in epithelial homeostasis.

In our interaction network, TP63 interconnected the highly connected regulators that are important for stratified epithelial development, proliferation, and differentiation (Fig. 2c). Interestingly, the motifs for stratification-related TFs, like TP63, KLF7, SOX9, KLF4, and KLF5 were concurrently enriched at SEs near their own loci (Fig. 2d and Supplementary Fig. 2a). These SE-regulated TFs formed an interconnected regulatory model that may balance stratified epithelial proliferation and differentiation.

Strikingly, our regulatory network model identified a cohort of interconnected auto-regulatory TF cliques termed CRCs that in turn co-regulated an extended SE network (Supplementary Fig. 2b). These CRCs consisted of distinct combinations of core TFs. Those key regulators important to corneal epithelial identity and homeostasis were embedded in these auto-regulatory cliques with high clique enrichment fractions (Fig. 2e). As expected, the CRC TFs exhibited strong network interconnectivity and cellular specification (Fig. 2b, e).

### RPS assemble a lineage-specific CRC

To understand the regulatory circuitries driving lineage determination of LSCs, we sought to identify potential core TFs. Total connectivity (in-degree + out-degree) predicted RUNX1, SMAD3, and FOSL2 as the ‘core’ of the TF networks (Fig. 2b). In addition, RUNX1 scored best in CRC clique enrichment and SMAD3 showed the highest expression level (Fig. 2e and Supplementary Fig. 2c). Thus, we focused on RUNX1 and phosphorylated SMAD3 (pSMAD3, the activated state of SMAD3), which were prominently expressed in human limbus and central corneal epithelium as well as in cultured primary LSCs (Fig. 2f and Supplementary Fig. 2d, e). Their expression patterns precisely mirrored that of PAX6. RUNX1 and SMAD3 were driven by SEs, and their motifs were also significantly enriched in SEs, TEs, and ATAC HSs (Fig. 2g and Supplementary Fig. 2f), allowing them to bind these regulatory elements. These data raised the possibility that RUNX1 and SMAD3 may be core regulators of the corneal epithelium.

We then dissected the binding profiles of endogenous RPS, and found that a large SE domain with high levels of H3K27ac, H3K4me1, and ATAC signals within the *PAX6* locus was co-occupied by RPS (Fig. 2h). Likewise, the SE elements within the *RUNX1* and *SMAD3* loci exhibited co-localization of RPS (Fig. 2h). In addition to forming this feed-forward auto-regulatory loop, RPS also co-regulated a subset of other SE domains including those close to key regulators of corneal epithelium and other pan-epithelial TFs, establishing an extended regulatory network (Fig. 2i and Supplementary Fig. 2g). These observations experimentally validated a lineage-specific CRC model established by RPS.

### The RPS-established CRC is required for LSC lineage commitment

**Fig. 3:**
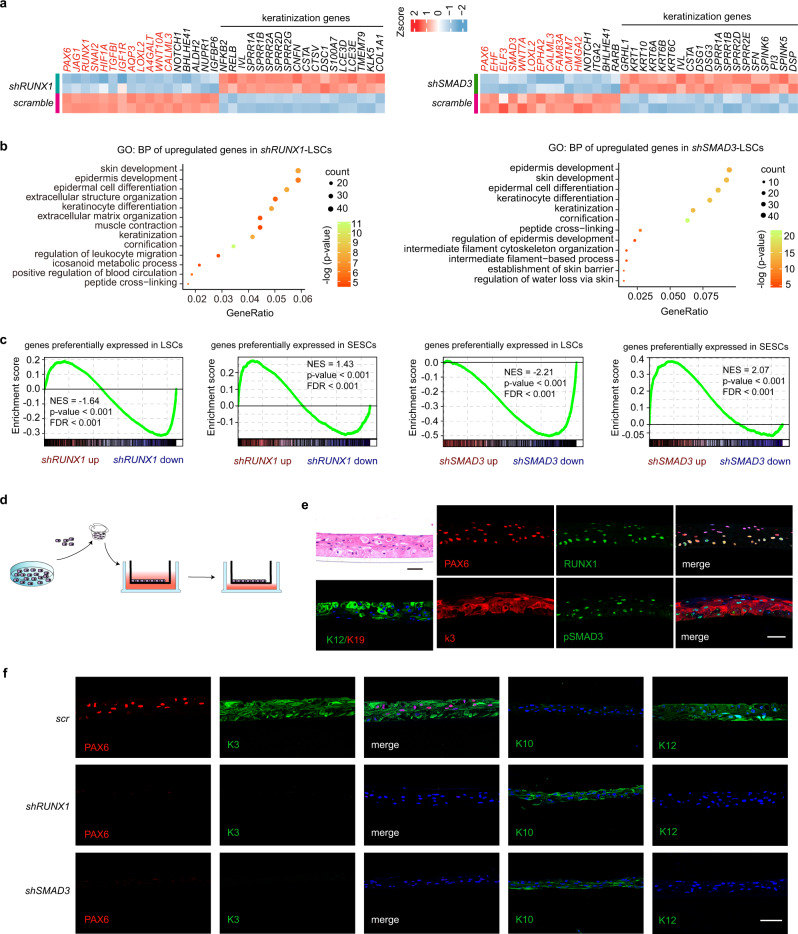
Loss of RUNX1 or SMAD3 induces cell identity switch. **a** Heatmaps of differentially expressed genes produced by *RUNX1* or *SMAD3* KD. Red mark represents SE-assigned genes. **b** GO BP analysis for the upregulated genes in *RUNX1*-depleted and *SMAD3*-depleted LSCs (pvalueCutoff = 0.01 and qvalueCutoff = 0.05). **c** GSEA for genes that (i) are expressed at higher levels in LSCs than in SESCs and (ii) are more highly expressed in SESCs than in LSCs. NES: normalized enrichment score. **d** Schema representation of the air-lifting culture system. LSCs were seeded in the transwell inserts and incubated in medium until they were confluent. Then, the medium in the upper chamber was removed to induce differentiation into a stratified epithelium sheet. **e** Hematoxylin and eosin (H&E) and immunofluorescence staining of the indicated genes in the differentiated corneal epithelium sheet after air-lifting induction. Scale bars, 50 μm. **f** Immunofluorescence staining of the indicated genes in the differentiated corneal epithelium sheets treated with the indicated shRNAs. Scale bar, 50 μm.

PAX6 has been identified as a core regulator of corneal epithelium fate determination in our previous publication^17^. To investigate the potential function of RUNX1 and SMAD3, small hairpin RNAs (shRNAs) were used to knock down (KD) their expression in LSCs (Supplementary Fig. 3a–c). Deficiency of either *RUNX1* or *SMAD3* remarkably reduced expression of *PAX6* and *NOTCH1* (Fig. 3a and Supplementary Fig. 3d), which are two key regulators for non-keratinized cell fate of the corneal epithelium^13,17^. RUNX1 also positively regulated many other key SE genes including *JAG1*, *TGFBI*, *IGF1R*, and *AQP3*, while SMAD3 activated *WNT7A* and *EHF* (Fig. 3a). A significant reduction in the expression of genes linked to biological processes for eye development, eye morphogenesis, cell fate commitment, and pan-epithelial identity were observed upon *RUNX1* depletion (Supplementary Fig. 3e). *SMAD3* KD decreased gene networks related to cell migration, wound healing, and TGFBR signaling pathway, as well as epithelial development and function (Supplementary Fig. 3e).

In contrast, the expression of a cluster of keratinization or cornification genes were induced by *RUNX1* or *SMAD3* KD (Fig. 3a). Importantly, upregulation of the key regulators of epidermal differentiation (*GRHL1* in *SMAD3*-KD LSCs, *NFKB2* and *RELB* in *RUNX1*-KD LSCs)^35,36^ occurred (Fig. 3a). As characterized by GO analysis, the up-regulated genes in either group displayed significant association with keratinization/cornification and epidermal development, differentiation, and function (Fig. 3b), which was strikingly similar to the results observed from skin epithelium stem/progenitor cell (SESC)-specific gene profile (Supplementary Fig. 3f). Gene set enrichment analysis (GSEA) also revealed overall transcriptome changes away from LSC transcripts and toward SESC transcripts after *RUNX1* or S*MAD3* depletion (Fig. 3c), suggesting that a transition to keratinized epidermal-like identity occurred.

Using the air-lifting culture system (Fig. 3d), LSCs were successfully differentiated into a multi-layered corneal epithelial sheet with strong expression of the CEC-specific markers KRT3 and KRT12 and no expression of the stem cell marker KRT19 (Fig. 3e and Supplementary Fig. 3g). In accordance with the results observed in human corneal tissues (Fig. 2f and Supplementary Fig. 2d), RPS were also detected in the in vitro-differentiated corneal epithelial sheet (Fig. 3e). Remarkably, upon in vitro differentiation, *RUNX1* or *SMAD3* KD in LSCs resulted in the differentiation into keratinized epidermal-like epithelial cells, as evidenced by the loss of PAX6, KRT3, and KRT12, and the appearance of keratinization marker KRT10 (Fig. 3f and Supplementary Fig. 3h). In contrast, overexpression of RUNX1 led to upregulation in the expression of *KRT3* and *KRT12*, while SMAD3 overexpression upregulated KRT3 expression (Supplementary Fig. 3i). We found that RUNX1 bound to the upstream enhancer of *KRT3* that was enriched for ATAC, H3K27ac, and H3K4me1 signals and occupied a downstream enhancer of *KRT12*. But the binding of SMAD3 across the *KRT3* and *KRT12* loci was not observed (Supplementary Fig. 3j). Altogether, we demonstrated that RUNX1 and SMAD3 are required for lineage commitment and identity of LSCs. *RUNX1-*depleted or *SMAD3-*depleted CECs exhibited epidermal-like features. Our results highlighted that CRC TFs control fate and plasticity of LSCs.

### Cooperative interaction of RPS specifies functional *cis*-regulatory atlas

**Fig. 4:**
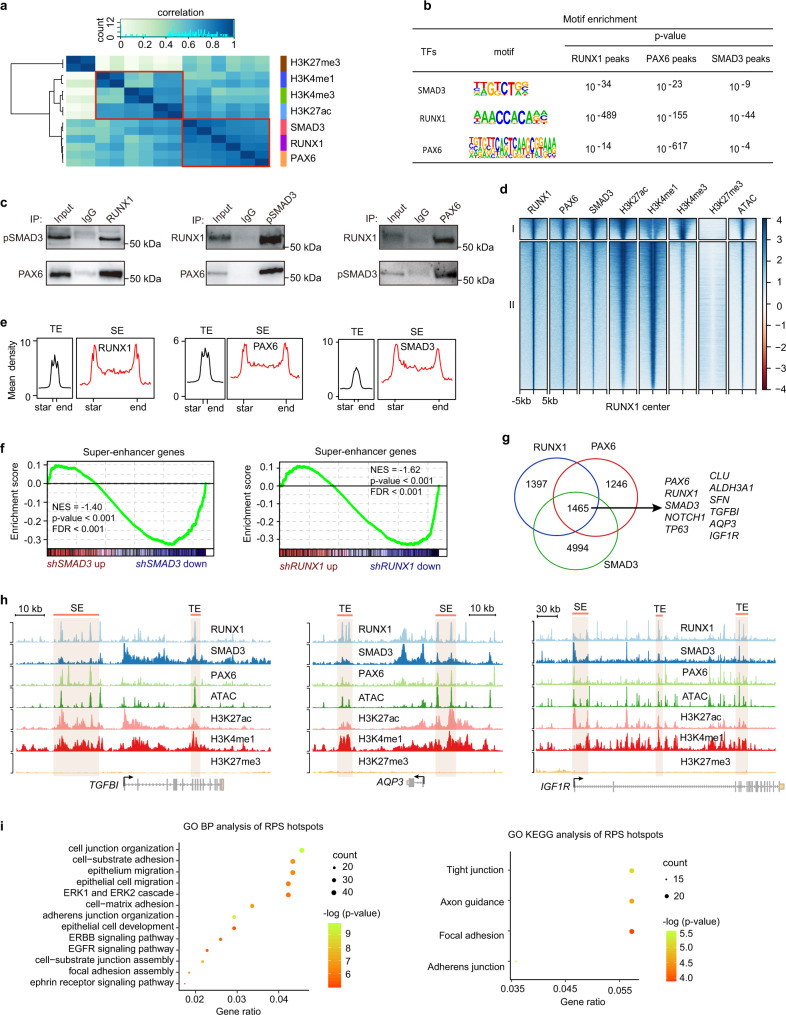
RPS complex positively regulates SE activity. **a** Hierarchical clustering of the indicated ChIP-seq-binding profiles using affinity (read count) scores. Pearson correlation coefficients between each group are shown. **b** Motif enrichment within RPS peaks in LSCs. **c** Co-immunoprecipitation analysis of the interactions among endogenous RUNX1, PAX6, and pSMAD3 in LSCs. **d** Heatmaps grouped into two clusters using *k*-means algorithm for the indicated ChIP-seq and ATAC-seq signals at RUNX1-bound sites. **e** Metaplots of average RPS ChIP-seq signals across TEs and SEs in LSCs. **f** GSEA of SE-associated gene set in scrambled shRNA-treated versus *shRUNX1*-treated LSCs and scrambled shRNA-treated versus *shSMAD3*-treated LSCs. **g** Venn diagram showing the overlapping and unique peaks bound by RPS. **h** Genome browser tracks for the indicated ChIP-seq and ATAC signals across the *TGFBI*, *AQP3*, and *IGF1R* loci. **i** GO BP and KEGG analysis for the genes closest to the overlapping RPS peaks with pvalueCutoff = 0.01 and qvalueCutoff = 0.05.

We found that the binding profiles of RPS were strongly correlated with each other and those of the active epigenetic signatures (Fig. 4a). Motifs for RPS were co-enriched at the centers of their respective peaks (Fig. 4b), indicative of their frequent binding in close proximity and functional cooperation. Furthermore, co-immunoprecipitation experiment verified that endogenous RUNX1, PAX6, and pSMAD3 proteins physically interacted with each other in LSCs (Fig. 4c).

We then dissected the distribution pattern of RPS complex across the genome. RPS-binding sites overlapped highly throughout the genome, which was exemplified by the SE loci (Fig. 2h and Supplementary Fig. 2g), and were grouped into active promoter and enhancer clusters (Fig. 4d). As RPS bound extensively to distal regions and their binding motifs were also frequently enriched at enhancers (Figs. 4d, 2g), we proposed that RPS complex defined a lineage-restricted enhancer repertoire. Indeed, both SEs and TEs were densely occupied by these three TFs (Fig. 4e and Supplementary Fig. 4a). Approximately 44.8% of all SEs were co-bound by RPS, and most (~78.6%) were occupied by at least two TFs, whereas only a small fraction of TEs were bound by ≥2 TFs (Supplementary Fig. 4b). Furthermore, GSEA revealed that the SE or TE gene set was only statistically significantly enriched in the *RUNX1* or *SMAD3* KD group (Fig. 4f and Supplementary Fig. 4c), underscoring that RUNX1 and SMAD3 positively regulated SE and TE activities.

RPS also co-localized at the accessible and active transcriptional start sites (TSSs; Supplementary Fig. 4d). In particular, a small portion of proximal promoters that were weakly bound by RPS exhibited the bivalent state (co-enrichment for H3K4me3, H3K27me3, and low H3K27ac and ATAC signal), while those promoters without RPS occupancy were inactive (Supplementary Fig. 4d). Thus, RPS complex may be associated with promoter activation.

TF hotspots, which are functional regulatory elements co-occupied cooperatively by multiple core TFs, specify cellular identity^37,38^. By intersecting RPS peaks, we found that the RPS co-binding hotspots were linked to many key corneal epithelial genes such as *CLU*, *ALDH3A1, NOTCH1, TP63*, and *SFN*^39^ as well as themselves (Fig. 4g). Specifically, RPS complex occupied SEs and TEs across the loci of *TGFBI*, *AQP3*, and *IGF1R* (Fig. 4h). In addition, RPS hotspot-associated genes were enriched for the fundamental pan-epithelial biological processes and signaling pathways (Fig. 4i). Interestingly, RPS hotspots contained the motifs for SE-driven corneal epithelial key TFs EHF, SOX9, KLF5, TP63, AP-1, and STAT3^40^ (Supplementary Fig. 4e), inferring their potential co-localization and functional synergy. These results further highlighted the importance of TF hotspots. Collectively, we showed that RPS cooperativity defines chromatin-regulatory architecture that confers lineage function and identity.

### RUNX1 establishes lineage-restricted epigenetic architecture via governing H3K27ac deposition

**Fig. 5:**
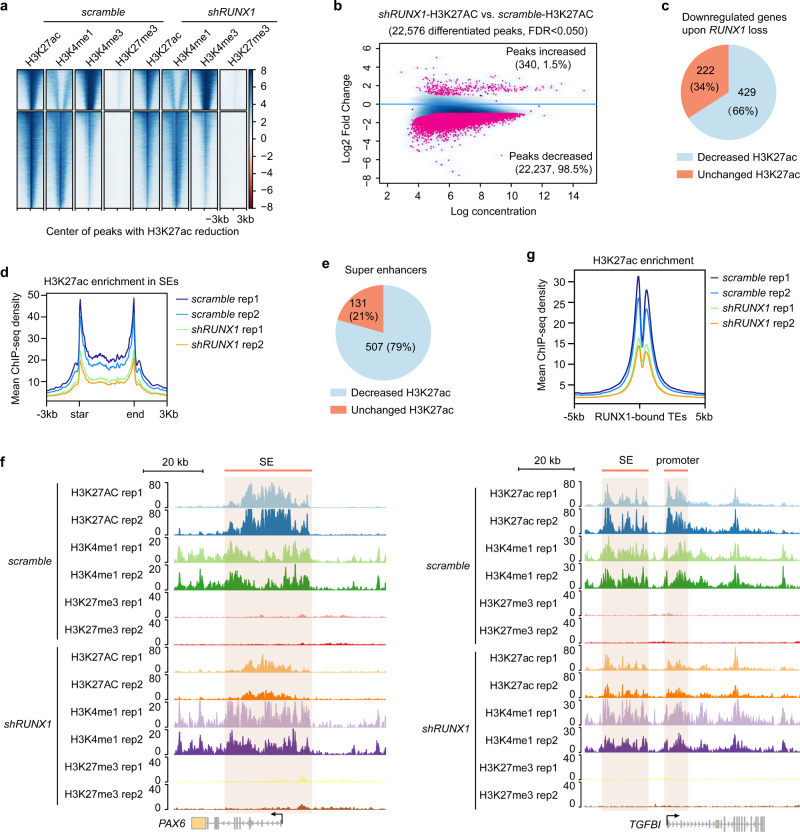
*RUNX1* depletion impairs H3K27ac deposition across lineage-specific enhancers. **a** Heatmaps showing the indicated ChIP-seq signals across the peaks with H3K27ac reduction in *shRUNX1*-treated versus scrambled shRNA-treated LSCs. **b** Scatterplot of H3K27ac enrichment in *shRUNX1*-treated versus scrambled shRNA-treated LSCs. Sites identified as significantly differentially bound (fold change ≥ 2, FDR < 0.05) are shown in red. **c** Pie chart showing the percentages of reduced and unchanged H3K27ac signals close to the loci of down-regulated genes upon *RUNX1* loss. **d** Metaplots of average H3K27ac density across the SEs of wild-type LSCs in *shRUNX1*-treated and scrambled shRNA-treated LSCs. **e** Pie chart showing the percentages of reduced and unchanged H3K27ac signals across the SEs of wild-type LSCs upon *RUNX1* loss. **f** Genome browser tracks for the indicated ChIP-seq signals across the *PAX6* and *TGFBI* loci in scrambled shRNA-treated and *shRUNX1*-treated LSCs. **g** Metaplots of average H3K27ac density across the RUNX1-binding TEs in scrambled shRNA-treated and *shRUNX1*-treated LSCs.

Maintenance of cell identity is strongly dependent on the specific chromatin environments that are established by lineage TFs to dominate transcriptional landscape^3,4^. To determine whether RUNX1 establishes a lineage-specific epigenetic landscape, we compared the binding maps of H3K27ac, H3K4me1, H3K4me3, and H3K27me3 in scrambled shRNA-treated and *RUNX1*-depleted LSCs. Negligible changes in the enrichment of H3K4me1, H3K4me3, and H3K27me3 throughout the genome were observed (Fig. 5a and Supplementary Fig. 5a). Intriguingly, 22,576 differential H3K27ac peaks (≥2-fold, false discovery rate [FDR] < 0.05) were identified upon *RUNX1* depletion, almost all of which (98.5%, 22,237/22,576) showed diminished H3K27ac deposition (Fig. 5b). The peaks with decreased H3K27ac included 4331 sites (19.5%) in proximal promoters and 17,906 sites (80.5%) in distal enhancers (Supplementary Fig. 5b). Combined with RNA-seq data, we found that 66% (429/651) of the genes transcriptionally downregulated after *RUNX1* KD were accompanied by reduction of H3K27ac signal close to their loci (Fig. 5c), suggesting that RUNX1 activated lineage-specific gene transcription by modulating H3K27ac deposition.

More importantly, the global H3K27ac signal across SE regions remarkably decreased in the *RUNX1*-depleted LSCs compared to that in the control group (Fig. 5d). Most SEs (79%, 507/638) exhibited significant reduction in H3K27ac level (Fig. 5e). In *RUNX1*-deficient LSCs, only 384 and 143 SEs were, respectively, identified by H3K27ac in two biological duplicates (Supplementary Fig. 5c), and these numbers were far fewer than those in wild-type LSCs (638 and 644). In particular, the dramatic reduction in H3K27ac level occurred in the RUNX1-bound SE domains across the *PAX6*, *TGFBI*, *AQP3*, *A4GALT*, and *WNT10A* loci upon *RUNX1* loss, whereas no evident changes in the enrichment of H3K4me1 and H3K27me3 were observed (Fig. 5f and Supplementary Fig. 5d). This resulted in the conversion of SEs into TEs, which was accompanied by the decrease in the transcription level of their target genes (Fig. 3a), suggesting that RUNX1 regulated their expression by directly activating the SE activities. Loss of *RUNX1* also diminished H3K27ac within its own SE domain (Supplementary Fig. 5d), indicative of an auto-regulatory circuitry. These data demonstrated that RUNX1 dominates the establishment of lineage-specific SEs.

The *RUNX1*-deficient LSCs also showed a significant reduction in the average H3K27ac level at RUNX1-bound TEs (Fig. 5g). Some TEs switched to primed enhancers characterized by high H3K4me1 enrichment and the lack of H3K27ac and H3K27me3 (Supplementary Fig. 6a), resulting in compromise of the corresponding transcription events (Fig. 3a). Given that RUNX1-mediated gene regulation in hematopoietic system requires interaction with histone acetyltransferase EP300, which acts as a co-activator^41,42^, we generated the binding map of EP300 in scrambled shRNA-treated and *shRUNX1*-treated LSCs. Indeed, we found that EP300 occupancy was dramatically affected at RUNX1-bound SEs and TEs with diminished H3K27ac levels upon *RUNX1* depletion (Supplementary Fig. 6b). We proposed that RUNX1 may recruit the epigenetic regulator EP300 to modulate H3K27ac deposition in LSCs. However, H3K27ac at SEs and TEs was not affected when SMAD3 was knocked down (Supplementary Fig. 6c). Our phenotypic and molecular data supported that RUNX1 governs the establishment of epigenetic landscape that defines corneal epithelial identity and homeostasis.

### Disruption of RPS cooperativity is linked to human corneal diseases

**Fig. 6:**
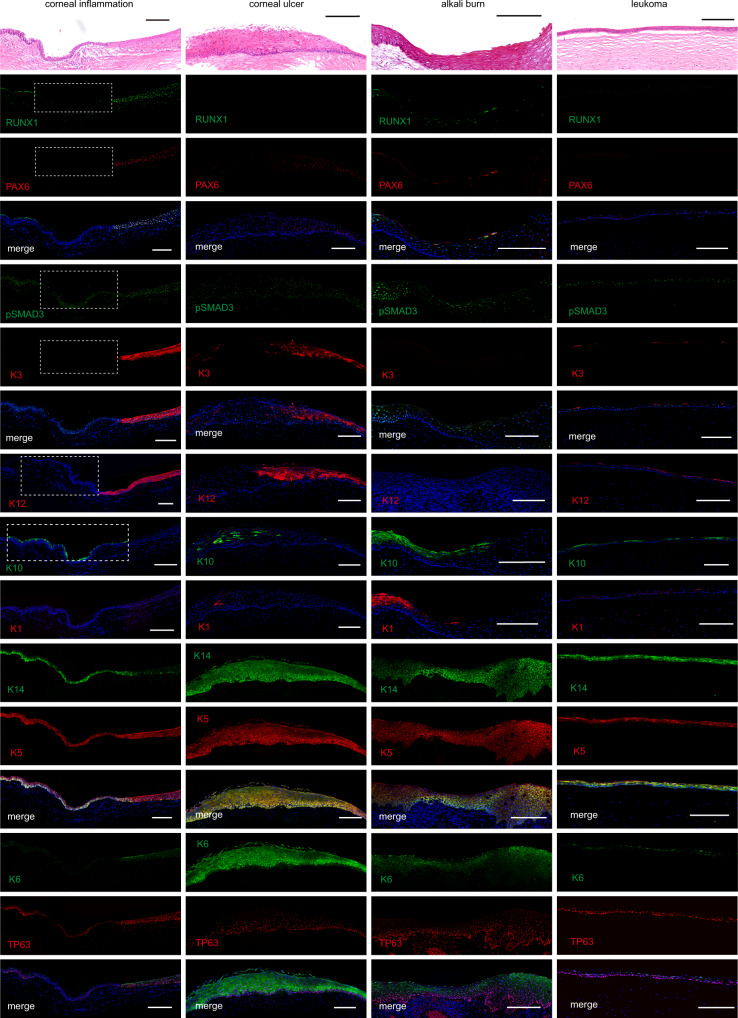
Pathological changes of corneal epithelium. H&E staining and immunofluorescence analysis of the indicated genes in corneal inflammatory (47 years old, female), ulcer (62 years old, female), alkali burn (33 years old, male), and leukoma (51 years old, female) tissues. Scale bars, 200 μm.

To further explore the potential role of RPS complex in corneal homeostasis in vivo, several types of common human corneal diseases, including inflammation, ulcer, alkaline burn, and leucoma were tested. We found that the expression of RUNX1, PAX6, and KRT12 was conspicuously lost in most areas of the lesioned tissues in all examined patients, and KRT10 and KRT1 (absent in the normal cornea) were detected in most of these areas instead (Fig. 6 and Supplementary Figs. 7–10, 11a, b). Interestingly, a partial area of the inflammatory corneal epithelial tissue retaining RUNX1 and pSMAD3 expression was PAX6/KRT3/KRT12-positive and KRT1/KRT10-negative (Fig. 6). All diseased corneal epithelial tissues showed expression of the stratified epithelial markers TP63, KRT14, and KRT5. The hyperproliferative and inflammatory state marker KRT6 was expressed in most of the patient samples (Fig. 6 and Supplementary Figs. 7–10, 11a). The gene expression patterns of these pathological corneal tissues were similar to those of the human epidermis (Supplementary Fig. 11c), indicating that the CECs of these diseased tissues switched to skin epidermal-like cells.

Of note, RUNX1 expression was extremely low in the basal cell layer of the human epidermis, whereas the conspicuous expression of pSMAD3 was observed in the epidermis and diseased corneal epithelial tissues (Fig. 6 and Supplementary Figs. 7–10, 11c). Thus, RUNX1 and PAX6 deficiency correlated highly with human corneal pathogenesis, and pSMAD3 alone in corneal lesions and epidermis is unable to maintain corneal epithelial identity due to the absence of its partners RUNX1 and PAX6.

## Discussion

**Fig. 7:**
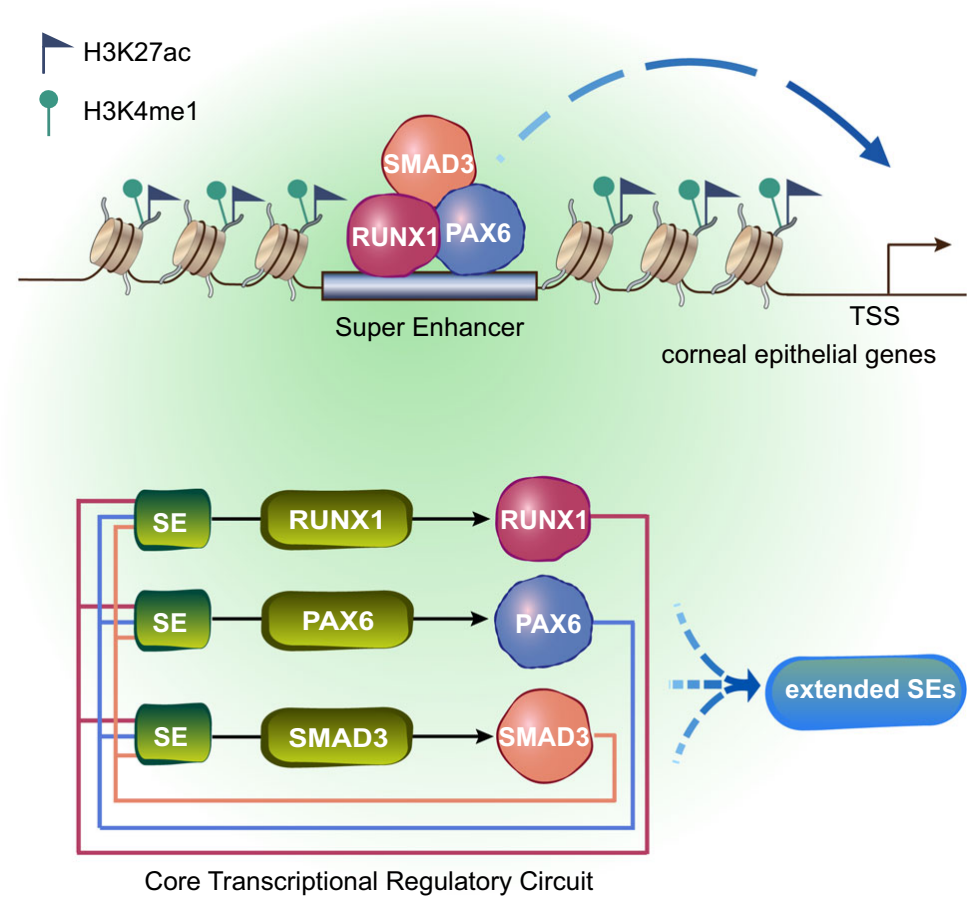
The CRC model of lineage-determining TFs underling corneal epithelial identity. RUNX1/PAX6/SMAD3 complex governs their own SEs and a broader SE network, establishing the active chromatin environment to maintain expression of the corneal epithelial identity genes.

The corneal epithelium shares high similarity with other stratified epithelia, especially in the balance between self-renewal and differentiation. In this study, we map the epigenetic landscape of LSCs and identify an RPS-mediated chromatin-regulatory networks required for corneal epithelial identity and homeostasis (Fig. 7). We find that the stratification-related TFs concurrently bind to their own SE domains, forming a comprehensive responsive circuitry to balance proliferation and differentiation of stem cells. As evidenced in mouse skin epithelium, differentiation factor Irf6 is activated directly by p63, which in turn induces p63 degradation to promote epidermal differentiation^34,43^. TEAD (a pro-proliferation factor) and KLF4 (a pro-differentiation factor) limit each other activity in epidermis and the balance of transcriptional activity between them is important for skin epithelial homeostasis^44^. Thus, we suggest that stem cell state dependents on the co-regulation of proliferation and differentiation TFs. We speculate that the unexplored TFs identified in our network analysis may be also crucial for stratified epithelia.

A major highlight of this work is that we integrate gene expression, chromatin accessibility, and TF–SE binding to define CRCs of LSCs. The CRCs recapitulate a set of TFs that have been demonstrated to play a key role in the corneal epithelium, such as PAX6, KLF4, SOX9, TP63, and MYC. SOX9 regulates proliferation and differentiation of LSCs^45,46^. KLF4 maintains corneal epithelial homeostasis by promoting epithelial cell fate and inhibiting epithelial–mesenchymal transition^11^. In particular, a combination of PAX6, KLF4, and OCT4 induces the expression of KRT3 and KRT12 in non-ocular epithelial cells^47^. Overexpression of OVOL2, PAX6, KLF4, SOX9, TP63, and MYC can reprogram fibroblasts into corneal epithelial-like cells^46^. These observations highlight that cooperation between the CRC TFs is critical to fate commitment of stem cells. Our findings reveal the coordinated regulatory model of these TFs.

Of note, the predicted TF-chromatin binding landscape reveal that RUNX1 is involved in the SE-mediated regulatory networks established by those well-known key TFs, forming the LSC-specific CRCs. Through functional validation, we find that RUNX1 is required for the maintenance of corneal epithelial identity and homeostasis. We also highlight that RUNX1 regulates H3K27ac deposition at the corneal epithelial key genes by recruiting EP300. Previous studies on RUNX1 were mainly concentrated on hematopoiesis and leukemia^48,49^. During hematopoiesis, RUNX1 interacts with epigenetic regulatory proteins, such as EP300, to regulate the expression of important hematopoietic genes through chromatin remodeling. Recently, a few insights into the role of RUNX1 in epithelial biology and pathology are unraveled^50^. For example, Runx1 modulates developmental hair follicle stem cell (HFSC) activation and promotes anagen onset and HFSC proliferation in adulthood^51^. Although we only observe the weak expression of RUNX1 in the basal cell layer of epidermis, there is significant evidence that RUNX1 is overexpressed and showed oncogene function in human skin, head and neck, and oral squamous cell carcinoma^50^. On the contrary, the limbus and corneal epithelium show robust expression of RUNX1 and loss of RUNX1 occurs in the lesions of the human corneal disease tissues. Thus, RUNX1 plays distinct roles in different tissues. In the corneal epithelium, RUNX1 maintains the epithelial cell fate during differentiation.

We also confirm protein–protein interactions among RPS, and highlight the importance of cooperative interaction of TFs in cell fate determination. It is the hotspots co-occupied by these three TFs that define the corneal epithelial identity. To be noted, PAX6 establishes distinct ventral progenitor cell populations and controls neuronal fate during development^52^. RUNX1 dominates stage-specific hematopoietic enhancer dynamics via interaction with alternate TFs at different stages of hematopoiesis^49^. Lineage-specific TFs direct distinct response of SMAD3 to external signals in various cells at different stages of development^53^. Thus, SMAD3 is unable to establish corneal-lineage transcription programs in the epidermis or corneal lesions due to deficiency of its partners RUNX1 and PAX6, whereas induction of PAX6 can convert SESCs to LSC-like cells^17^. Taken together, we identify RUNX1 and SMAD3 as two corneal lineage core regulators that establish LSC-specific epigenetic atlas. The transcriptional regulatory network uncovered by our study provides major insights into the identity, lineage commitment and plasticity of adult stem cell.

Precise medicine shapes the future of basic and medical study. Despite the challenges, scientists are making an effort to drive the ongoing advances in gene therapy for human diseases. A variety of clinical trials and preclinical studies have verified that Clustered Regularly Interspaced Short Palindromic Repeat/Cas9 (CRISPR/Cas9)-mediated somatic gene editing has the potential for therapeutic applications in humans^54,55^. Gene delivery into somatic cells especially adult stem/progenitor cells can result in significant functional improvement in human skin, retina, and chronic granulomatous diseases^55–57^. The functional importance of RUNX1 and PAX6 in corneal homeostasis and their loss in corneal pathology imply their potential clinical applications of corneal regenerative therapies.

## Methods

### Human samples

All human pathological cornea and normal limbus were provided as de-identified surgical specimens. Supplementary Fig. 11a lists the age and gender of each patient donor of diseased corneal tissues. Normal skin biopsies were obtained from eye lips of donors. All human tissues were obtained with the approval of the Ethics Committee of Zhongshan Ophthalmic Center of Sun Yat-sen University.

### Primary cell culture

Human LSCs and SESCs were isolated from donors and cultured in feeder-free plates as previously described^17^. Briefly, the limbus tissues were isolated from postmortem human eyeballs and cut into small pieces, followed by digestion with 0.2% collagenase IV (Gibco) at 37 °C for 2 h and further digestion using 0.25% trypsin–EDTA (Gibco) at 37 °C for 15 min. Then, the cells were seeded and cultured on polystyrene plates (Corning) coated with 2% Matrigel (BD Biosciences). The components of LSC medium included DMEM/F12 and DMEM (1:1) with 1% penicillin/streptomycin, 10% fetal bovine serum, 10 ng/ml EGF, 5 μg/ml insulin, 0.4 μg/ml hydrocortisone, 0.1 nM cholera toxin, and 2 nM 3,3′,5-triiodo-l-thyronine. Human epidermal tissues were obtained from eye lids, cut into small pieces, digested, and seeded on coated polystyrene plates following the above steps. SESCs were cultured using LSC medium.

### Air-lifting culture system

Cultured LSCs were seeded on 2% Matrigel-coated transwell inserts (0.4 μm polyester membrane, Corning) in 24-well culture dishes. Cells were incubated with medium both in inserts (200 μl) and lower dishes (1 ml) until confluent. To promote differentiation, medium in the upper storey of the chamber was removed to expose the cells to the air and the lower compartment remained filled with medium (200 μl, medium was changed every day) for additional 5–8 days. The medium used in air-lifting system was LSC medium.

### Gene knockdown and RNA-seq assays

shRNAs targeting *RUNX1* and *SMAD3* were cloned into PLKO.1 plasmid. Scrambled shRNA from Addgene that does not target any known genes in human genome was used as control. Two target-specific shRNAs were used for either gene knockdown. Two shRNAs against a target were used separately. LSCs were infected with lentivirus particles encoding specific shRNA for 24 h, and positive cells were selected with 2 μg/ml puromycin for 48 h. Total RNA was extracted from cells using the RNeasy Mini kit (Qiagen) and cDNA was synthesized using the PrimeScript™ RT Master Mix kit (Takara). Real-time quantitative PCR was performed with the iTaq™ Universal SYBR^®^ Green Supermix kit (Bio-Rad). Gene expressions were normalized to that of GAPDH. The primers used in this paper is provided in Supplementary Table 1. shRNAs used are as follows:

scramble: 5′-CCTAAGGTTAAGTCGCCCTCG-3′;

*shSMAD3*-1: 5′-GAGCCTGGTCAAGAAACTCAA-3′;

*shSMAD3*-2: 5′-CCGCTGTTCCAGTGTGTCTTA-3′;

*shRUNX1*-1: 5′-CCTCGAAGACATCGGCAGAAA-3′;

*shRUNX1*-2: 5′-GCTGAGAAATGCTACCGCAGC-3′.

For cDNA library construction, total RNAs were sheared to fragments of ~250 bp and the fragments were reverse transcribed through the NEBNext RNA first and second Strand Synthesis Module (NEB, USA). After end repair, A-tailing, adapter ligation, and amplification (KAPA Library Preparation Kit, Kapa Biosystem), the products were sequenced on an Illumina HiSeq X-Ten instrument with paired-end 150 reads setting.

### H&E staining and immunofluorescence (IF)

Tissue samples were fixed in 10% neutral buffered formalin solution for 1 h at room temperature, followed by dehydration and paraffin embedding. For H&E staining, after de-paraffinization, the tissue sections were stained with hematoxylin and eosin. Cells were fixed for 15 min with 4% paraformaldehyde at room temperature.

For IF, de-paraffinization of paraffin-embedded tissue section was performed, followed by permeabilization and blocking using PBS solution containing 0.3%Triton X-100 and 3% BSA for 1 h. Next, the sections were immunostained with primary antibodies overnight at 4 °C and were sequentially incubated with secondary antibodies for 1 h, followed by counterstaining with DAPI (4′,6-diamidino-2-phenylindole). Slides were imaged using ZEISS LSM 800 confocal laser scanning microscope.

The antibodies used for IF are as follows: anti-PAX6 (BioLegend, 901301,1:500), anti-PAX6 (Sigma, AMAB91372,1:500), anti-RUNX1 (Abcam, ab23980,1:500), anti-pSMAD3 (Abcam, ab52903,1:500), anti-K19 (Thermo Scientific, MS-1902-P,1:1000), anti-p63 (BioLegend, 619002,1:500), anti-Ki67 (CST, 9129S,1:500), anti-Cytokeratin 1 (Invitrogen, MA1-06312, 1:1000), anti-Cytokeratin 10 (Invitrogen, MA1-06319, 1:1000), anti-Cytokeratin 3 (Abcam, ab68260, 1:1000), anti-Keratin 12 (Abcam, ab124975, 1:1000), anti-rabbit IgG (Alexa Fluor 488 Conjugate, CST, 4412S, 1:1000), anti-mouse IgG (Alexa Fluor 488 Conjugate, CST, 4408S, 1:1000), anti-rabbit IgG (Alexa Fluor 594 Conjugate, CST, 8889S, 1:1000), and anti-mouse IgG (Alexa Fluor 594 Conjugate, CST, 8890S, 1:1000).

### ChIP-seq

Cells were crosslinked with 1% formaldehyde for 10 min at room temperature and lysed in sonication buffer (50 mM HEPES–NaOH, pH 7.5, 500 mM NaCl, 1 mM EDTA, 0.1% Na-deoxycholate, 1% TritonX-100, and 0.1% SDS). Sonication was performed using Covaris M220 focused-ultrasonicator to shear crosslinked chromatin. Then, chromatin extract was incubated in IP buffer (HEPES–NaOH, pH 7.5, 300 mM NaCl, 1 mM EDTA, 0.1% Na-deoxycholate, 1% TritonX-100, and 0.1% SDS) with primary antibodies overnight at 4 °C, followed by addition of Protein G and Protein A Dynabeads (1:1, Invitrogen). The beads were sequentially washed with sonication buffer, low-salt wash buffer (10 mM Tris–HCl, pH 8.0, 250 mM LiCl, 1 mM EDTA, 0.5% NP40, and 0.5% Na-deoxycholate) and TE buffer (10 mM Tris–HCl, pH 8.0, and 1 mM EDTA).

Immunoprecipitated complex was eluted from beads and de-crosslinked using elution buffer (50 mM Tris–HCl, pH 8.0, 10 mM EDTA, and 1% SDS) at 65 °C for 4 h. The elute was treated with proteinase K (Invitrogen) and RNase A (Invitrogen) for 1 h and then purified using the MinElute PCR Purification Kit (Qiagen). Purified DNA was used to prepare ChIP-seq DNA libraries for Illumina sequencing using KAPA Hyper Prep Kit (Kapa Biosystems, KK8502). Library DNA was sequenced with paired-end 150 reads using an Illumina HiSeq X10 instrument.

The antibodies used for ChIP-seq (5 μg/ChIP) are as follows: anti-H3K27ac (Millipore, 07-360), anti-H3K27me3 (CST, 9733s), anti-H3K4me1 (Active Motif, 39297), anti-H3K4me3 (CST, 9751S), anti-PAX6 (BioLegend, 901301), anti-RUNX1 (Abcam, ab23980), anti-SMAD3 (Abcam, ab28379), and anti-EP300 (Abcam, ab14984).

### ATAC-seq

Adherent LSCs were digested with 0.25% trypsin. After washing with PBS, 50,000 cells were collected and lysed in ice-cold lysis buffer (10 mM Tris–HCl, pH 7.5, 10 mM NaCl, 3 mM MgCl_2_, 0.5% IGEPAL CA-630, and 0.1% Tween-20) for 5 min. Immediately after the nuclei were collected, Tn5 transposase reactions (Vazyme Biotech, TD501) were performed at 900 rpm and 37 °C for 30 min. The transposed DNA fragments were purified using the Qiagen MinElute PCR Purification Kit. Then, the purified DNA fragments were amplified using the TruePrep DNA Library Prep Kit (Vazyme Biotech) according to the manufacturer’s instructions. Next, Agencourt AMPure XP (Beckman) was used to enrich the amplified DNA library. Finally, library DNA was sequenced with paired-end 150 reads with an Illumina HiSeq X10 instrument.

### Co-immunoprecipitation and western blot

Cells were collected and resuspended in ice-cold hypotonic buffer (10 mM HEPES, pH 7.9, 1.5 mM MgCl_2_, 10 mM KCl, 0.5 mM DTT, 0.2 mM PMSF, and protease inhibitor cocktail) for 15 min. Then, IGEPAL CA-630 (final concentration 0.3%) was added into the cell suspension and the suspension was incubated for 5 min, following which nuclei were extracted. After one wash with hypotonic buffer, the nuclei were treated with nuclear extract buffer (20 mM HEPES, pH 7.5, 25% glycerol, 0.42 M NaCl, 1.5 mM MgCl_2_, 0.2 mM EDTA, 0.5 mM DTT, 0.2 mM PMSF, and protease inhibitor cocktail) for 30 min to release nuclear proteins. In order to dilute salt concentration to 150 mM, IP dilution buffer (20 mM HEPES, pH = 7.5, 18% glycerol, 1.5 mM MgCl_2_, 0.2 mM EDTA, 0.5 mM DTT, 0.2 mM PMSF, protease inhibitor cocktail, and 0.1% IGEPAL CA-630) was added to the nuclear protein extract (1.8:1). Next, target proteins were immunoprecipitated via incubation with primary antibodies overnight at 4 °C, followed by capture of Protein A/G Dynabeads. Protein complex was eluted and denatured using NuPAGE™ LDS Sample Buffer (Invitrogen). Protein interaction was analyzed by western blotting as previously described^17^. The full scans blots were provided in the Source Data file.

The antibodies used for immunoprecipitation (IP, 10 μg) and western blot (WB,1:1000) are as follows: anti-PAX6 (IP, BioLegend, 901301), anti-PAX6 (WB, Invitrogen, PA1-801), anti-RUNX1 (IP, Abcam, ab23980), anti-RUNX1 (WB, BioLegend, 659302), anti-SMAD3 (WB, Invitrogen, MA5-15663), and anti-pSMAD3 (IP, Abcam, ab52903).

### Data analysis of ChIP-seq and ATAC-seq

For ChIP-seq and ATAC-seq data, reads were trimmed using trimmomatic tool^58^ and then were aligned to human hg19 reference genome using BWA software (version 0.7.17)^59^. Duplicated reads were removed using the Picard Markduplicates (version 2.18.16; http://broadinstitute.github.io/picard/) and only uniquely mapping reads were retained for further analysis.

MACS2 (version 2.1.1)^60^ was used to call peaks. For sharp peaks, we used the following parameters: -f BAMPE -B–SPMR -q 0.001–call-summits–fix-bimodal–seed 11521–extsize 200. For broad peaks (H3K27me3 and H3K4me1), the following parameters were used: -f BAMPE -B–SPMR–fix-bimodal–extsize 500–broad–broad-cutoff 0.1–seed 11521. For ATAC peaks, the following parameters were used: -f BAMPE -B–SPMR -q 0.001–call-summits–seed 11521–nomodel–shift -100–extsize 200. To filter out the low signal regions, ATAC peaks only with pileup ≥40 were retained for further study.

Pearson’s correlation coefficient analysis based on the read coverages for genomic regions for BAM files was performed using deepTools multiBamSummary. Two independent biological replicates showed a high degree of similarity. A list of overlapping peaks between two biological replicates was generated by the HOMER mergePeaks command. Peak distributions were analyzed by ChIPseeker package^61^. The HOMER’s annotatePeaks.pl program^62^ was used for peak annotations with the default settings. BEDTools intersect was used for statistical analysis of overlap ratio between two sets of genomic features.

To visualize continuous ChIP-seq signal, bigwig files normalized to 1× genome coverage in 50 bp bins were created by deepTools bamCoverage (version 3.0.2). For ATAC bigwig files, we used an additional parameter:–Offset 1 to obtain the cleavage sites. The visualization of bigwig files was achieved using the plotTracks function in Gviz package. Heatmaps and profiles of both ChIP-seq and ATAC-seq signal were plotted using deepTools.

### Differential binding and correlation analysis of ChIP-seq data

DiffBind package^63^ in R environment with the default parameters was used to identify differential peaks between two sample groups based on two biological replicates for each sample. Only differential binding peaks with fold change ≥2 and FDR < 0.05 were used in further study. Correlation heatmap based on the affinity scores for ChIP-seq samples was also plotted using DiffBind package with the default settings.

### Super-enhancer identification

ChIP-seq signal of H3K27ac was used to define SEs. SEs were identified and annotated using the default parameters according to ROSE algorithm (https://bitbucket.org/young_computation/rose)^20^.

### Computational reconstruction of CRCs

The coltron python package (https://pypi.org/project/coltron/)^6^ was used to analyze SE-mediated CRCs according to a previously reported method^5,6^. Briefly, the interaction network of all SE-assigned TFs was constructed by inward and outward degrees that quantify connectivity among all nodes. Inward degree of node TF_x_ represented the total number of node TFs, of which motifs were enriched within TF_x_ proximal SE. Outward degree of TF_x_ represented the total number of node genes containing a proximal SE with TF_x_ motifs. The overlapped regions between ATAC peaks and H3K27ac-defined SEs were used for motif discovery using coltron. In this regulatory model, the interconnected autoregulatory networks (clique) consisting of distinct TF combinations are defined as CRCs. In the CRC TF cliques, each member binds its own SE and the SEs of the other TFs in the circuit and they also co-drive an extended SE catalog.

### RNA-Seq data analysis

For RNA-seq data, trimmed reads were aligned to human hg19 reference genome with STAR software (version 2.6.1a)^64^ to acquire read counts within each gene. The SAM files were converted into BAM format using SAMtools (version 1.9)^65^. TPM values that represent gene expression were generated using RSEM tools (version v1.3.0)^66^. Significantly differentially expressed genes with fold change ≥2 and FDR < 0.05 were determined using DESeq2 (version 1.20.0)^67^.

### Motif analysis

HOMER’s findMotifsGenome.pl program^62^ was used for motif analysis with the default parameters. Due to H3K27ac flanking the transcription factor-binding sites, motifs enriched in TEs were identified from ±1 kb sequences around its peak centers. For motif enrichment in SEs, we first intersected H3K27ac peak files with SE bed files to obtain overlapping peaks. Then, motifs were analyzed on ±1K sequences around the overlapping peak centers. Motifs enriched in TF peaks were found on ±100 bp sequences around the peak centers. Motifs enriched in ATAC peaks were found on ±50 bp sequences around peak centers.

### Gene ontology analysis

R package clusterprofiler^68^ was used for identification of significantly enriched GO biological processes and KEGG pathways with pvalueCutoff = 0.01 and qvalueCutoff = 0.05.

### Gene set enrichment analysis

GSEA^69^ was performed using official software downloaded from https://www.broadinstitute.org/gsea/ with weighted enrichment statistic and signal2noise ranking metric.

### Bioinformational illustration plotting

The bubble plots of GO analysis, bar plots, scatterplots, and boxplots were plotted using on-line imageGP (http://www.ehbio.com/ImageGP/index.php/Home/Index/index.html). Heatmaps of gene expression were plotted using “pheatmap” R package. The interaction networks were showed via cytoscape.

### Statistics and reproducibility

Statistical measurements were performed using GraphPad. Differences between groups were assayed using unpaired two-tailed Student’s *t*-test. All error bars were calculated in GraphPad and data were shown as the mean ± SD. In all figure legends, *n* value represents the number of independent biological replicates. Shown IF images of cells and normal tissues were representative of at least three independent biological experiments. IF staining of each type of the corneal diseases reported in this paper was performed in three patients.

### Reporting summary

Further information on research design is available in the Nature Research Reporting Summary linked to this article.

## Supplementary information

Supplementary Information

Reporting Summary

## Data Availability

All data supporting the findings of this study are available within the paper and its supplementary information file. All sequencing data are available through the following Gene Expression Omnibus accession number: GSE156273. Other relevant data are available from the corresponding authors upon reasonable request. Source data are provided with this paper.

## Source data

Source Data

## Code availability

The code used for super-enhancer identification are available at https://bitbucket.org/young_computation/rose^20^. The code used for CRC analysis are available at https://pypi.python.org/pypi/coltron^6^.

## References

[1] Blanpain C, Horsley V, Fuchs E (2007). Epithelial stem cells: turning over new leaves. Cell.

[2] Heinz S (2010). Simple combinations of lineage-determining transcription factors prime-regulatory elements required for macrophage and B cell Identities. Mol. Cell.

[3] Adam RC (2015). Pioneer factors govern super-enhancer dynamics in stem cell plasticity and lineage choice. Nature.

[4] Adam RC (2018). Temporal layering of signaling effectors drives chromatin remodeling during hair follicle stem cell lineage progression. Cell Stem Cell.

[5] Saint-André V (2016). Models of human core transcriptional regulatory circuitries. Genome Res..

[6] Lin CY (2016). Active medulloblastoma enhancers reveal subgroup-specific cellular origins. Nature.

[7] Ott CJ (2018). Enhancer architecture and essential core regulatory circuitry of chronic lymphocytic leukemia. Cancer Cell.

[8] Nowell, C. S. & Radtke, F. Corneal epithelial stem cells and their niche at a glance. *J. Cell Sci*. 198119 (2017). 10.1242/jcs.198119 28202689

[9] Thoft RA, Friend J (1983). The X, Y, Z hypothesis of corneal epithelial maintenance. Investig. Ophthalmol. Vis. Sci..

[10] Pellegrini G, De Luca M (2014). Eyes on the prize: limbal stem cells and corneal restoration. Cell Stem Cell.

[11] Tiwari A, Loughner CL, Swamynathan S, Swamynathan SK (2017). KLF4 plays an essential role in corneal epithelial homeostasis by promoting epithelial cell fate and suppressing epithelial–mesenchymal transition. Investig. Ophthalmol. Vis. Sci..

[12] Kitazawa K (2017). PAX6 regulates human corneal epithelium cell identity. Exp. Eye Res..

[13] Vauclair S (2007). Corneal epithelial cell fate is maintained during repair by Notch1 signaling via the regulation of vitamin A metabolism. Dev. Cell.

[14] Blanpain C, Fuchs E (2007). p63: revving up epithelial stem-cell potential. Nat. Cell Biol..

[15] Senoo M, Pinto F, Crum CP, McKeon F (2007). p63 is essential for the proliferative potential of stem cells in stratified epithelia. Cell.

[16] Koster MI, Kim S, Mills AA, DeMayo FJ, Roop DR (2004). p63 is the molecular switch for initiation of an epithelial stratification program. Genes Dev..

[17] Ouyang H (2014). WNT7A and PAX6 define corneal epithelium homeostasis and pathogenesis. Nature.

[18] Buenrostro JD, Giresi PG, Zaba LC, Chang HY, Greenleaf WJ (2013). Transposition of native chromatin for fast and sensitive epigenomic profiling of open chromatin, DNA-binding proteins and nucleosome position. Nat. Methods.

[19] Shlyueva D, Stampfel G, Stark A (2014). Transcriptional enhancers: from properties to genome-wide predictions. Nat. Rev. Genet..

[20] Whyte WA (2013). Master transcription factors and mediator establish super-enhancers at key cell identity genes. Cell.

[21] Hnisz D (2013). Super-enhancers in the control of cell identity and disease. Cell.

[22] Wakefield LM, Stuelten C (2007). Keeping order in the neighborhood: new roles for TGFP in maintaining epithelial homeostasis. Cancer Cell.

[23] Elbediwy A, Thompson BJ (2018). Evolution of mechanotransduction via YAP/TAZ in animal epithelia. Curr. Opin. Cell Biol..

[24] Gen L (2015). Transcription factor PAX6 (paired Box 6) controls limbal stem cell lineage in development and disease. J. Biol. Chem..

[25] Titone R, Zhu M, Robertson DM (2019). Mutual regulation between IGF-1R and IGFBP-3 in human corneal epithelial cells. J. Cell. Physiol..

[26] Verkman AS, Ruiz-Ederra J, Levin MH (2008). Functions of aquaporins in the eye. Prog. Retin. Eye Res..

[27] Lakshminarayanan R (2014). Clinical and genetic aspects of the TGFBI-associated corneal dystrophies. Ocul. Surf..

[28] Kheir V, Cortes-Gonzalez V, Zenteno JC, Schorderet DF (2019). Mutation update: TGFBI pathogenic and likely pathogenic variants in corneal dystrophies. Hum. Mutat..

[29] Jacobs J (2018). The transcription factor grainy head primes epithelial enhancers for spatiotemporal activation by displacing nucleosomes. Nat. Genet..

[30] Werth M (2010). The transcription factor grainyhead-like 2 regulates the molecular composition of the epithelial apical junctional complex. Development.

[31] Ge Y (2017). Stem cell lineage infidelity drives wound repair and cancer. Cell.

[32] Klein RH, Andersen B (2015). Dynamic networking for epidermal differentiation. Dev. Cell.

[33] Rubin AJ (2017). Lineage-specific dynamic and pre-established enhancer-promoter contacts cooperate in terminal differentiation. Nat. Genet..

[34] Richardson RJ (2006). Irf6 is a key determinant of the keratinocyte proliferation-differentiation switch. Nat. Genet..

[35] Pasparakis M (2002). TNF-mediated inflammatory skin disease in mice with epidermis-specific deletion of IKK2. Nature.

[36] Wilanowski T (2008). Perturbed desmosomal cadherin expression in grainy head-like 1-null mice. EMBO J..

[37] Liu Z (2014). Enhancer activation requires trans-recruitment of a mega transcription factor complex. Cell.

[38] Siersbaek R (2014). Transcription factor cooperativity in early adipogenic hotspots and super-enhancers. Cell Rep..

[39] Lu Q, Xin Y, Ye F, Foulks G, Li Q (2011). 14-3-3sigma controls corneal epithelium homeostasis and wound healing. Investig. Ophthalmol. Vis. Sci..

[40] Nakamura T (2014). LRIG1 inhibits STAT3-dependent inflammation to maintain corneal homeostasis. J. Clin. Investig..

[41] Yamaguchi Y (2004). AML1 is functionally regulated through p300-mediated acetylation on specific lysine residues. J. Biol. Chem..

[42] Kitabayashi I, Yokoyama A, Shimizu K, Ohki M (1998). Interaction and functional cooperation of the leukemia-associated factors AML1 and p300 in myeloid cell differentiation. Embo J..

[43] Moretti F (2010). A regulatory feedback loop involving p63 and IRF6 links the pathogenesis of 2 genetically different human ectodermal dysplasias. J. Clin. Investig..

[44] Yuan Y (2020). YAP1/TAZ-TEAD transcriptional networks maintain skin homeostasis by regulating cell proliferation and limiting KLF4 activity. Nat. Commun..

[45] Menzel-Severing J (2018). Transcription factor profiling identifies Sox9 as regulator of proliferation and differentiation in corneal epithelial stem/progenitor cells. Sci. Rep.-Uk..

[46] Kitazawa K (2016). OVOL2 maintains the transcriptional program of human corneal epithelium by suppressing epithelial-to-mesenchymal transition. Cell Rep..

[47] Sasamoto Y (2016). PAX6 isoforms, along with reprogramming factors, differentially regulate the induction of cornea-specific genes. Sci. Rep..

[48] Pulikkan JA (2018). CBFbeta-SMMHC inhibition triggers apoptosis by disrupting MYC chromatin dynamics in acute myeloid leukemia. Cell.

[49] Lichtinger M, Hoogenkamp M, Krysinska H, Ingram R, Bonifer C (2010). Chromatin regulation by RUNX1. Blood Cells Molecules Dis..

[50] Scheitz CJF, Tumbar T (2013). New insights into the role of Runx1 in epithelial stem cell biology and pathology. J. Cell. Biochem..

[51] Osorio KM (2008). Runx1 modulates developmental, but not injury-driven, hair follicle stem cell activation. Development.

[52] Ericson J (1997). Pax6 controls progenitor cell identity and neuronal fate in response to graded Shh signaling. Cell.

[53] Mullen AC (2011). Master transcription factors determine cell-type-specific responses to TGF-beta signaling. Cell.

[54] Doudna JA (2020). The promise and challenge of therapeutic genome editing. Nature.

[55] Lipinski DM, Thake M, MacLaren RE (2013). Clinical applications of retinal gene therapy. Prog. Retin. Eye Res..

[56] Hirsch T (2017). Regeneration of the entire human epidermis using transgenic stem cells. Nature.

[57] Kohn DB (2020). Lentiviral gene therapy for X-linked chronic granulomatous disease. Nat. Med..

[58] Bolger AM, Lohse M, Usadel B (2014). Trimmomatic: a flexible trimmer for Illumina sequence data. Bioinformatics.

[59] Li H, Durbin R (2010). Fast and accurate long-read alignment with Burrows–Wheeler transform. Bioinformatics.

[60] Zhang Y (2008). Model-based analysis of ChIP-Seq (MACS). Genome Biol..

[61] Chen TW (2014). ChIPseek, a web-based analysis tool for ChIP data. BMC Genomics.

[62] Heinz S (2010). Simple combinations of lineage-determining transcription factors prime cis-regulatory elements required for macrophage and B cell identities. Mol. Cell.

[63] Wu DY, Bittencourt D, Stallcup MR, Siegmund KD (2015). Identifying differential transcription factor binding in ChIP-seq. Front. Genet..

[64] Dobin A (2013). STAR: ultrafast universal RNA-seq aligner. Bioinformatics.

[65] Li H (2009). The Sequence Alignment/Map format and SAMtools. Bioinformatics.

[66] Li B, Dewey CN (2011). RSEM: accurate transcript quantification from RNA-Seq data with or without a reference genome. BMC Bioinforma..

[67] Love MI, Huber W, Anders S (2014). Moderated estimation of fold change and dispersion for RNA-seq data with DESeq2. Genome Biol..

[68] Yu G, Wang LG, Han Y, He Q (2012). Y. clusterProfiler: an R package for comparing biological themes among gene clusters. Omics.

[69] Subramanian A (2005). Gene set enrichment analysis: a knowledge-based approach for interpreting genome-wide expression profiles. Proc. Natl Acad. Sci. USA.

