# Supplementary material for: Core transcription regulatory circuitry orchestrates corneal epithelial homeostasis
Source: Nat Commun. 2021 Jan 18;12:420. doi: 10.1038/s41467-020-20713-z (PMC7814021; doi:10.1038/s41467-020-20713-z)
Supplement: Supplementary file 1 — Supplementary Information [file 41467_2020_20713_MOESM1_ESM.pdf]

Supplementary Information for

Core transcription regulatory circuitry orchestrates corneal epithelial homeostasis

Mingsen Li[1], Huaxing Huang[1], Lingyu Li[1], Chenxi He[2], Liqiong Zhu[1], Huizhen Guo, Li Wang[1], Jiafeng Liu[1], Siqi Wu[1], Jingxin Liu[3], Tao Xu[4], Zhen Mao[1], Nan Cao[4], Kang Zhang[1], Fei Lan[2], Junjun Ding[3], Jin Yuan[1], Yizhi Liu[1,5*] and Hong Ouyang [1,6,*]

*Correspondence: yzliu62@yahoo.com, Ouyhong3@mail.sysu.edu.cn

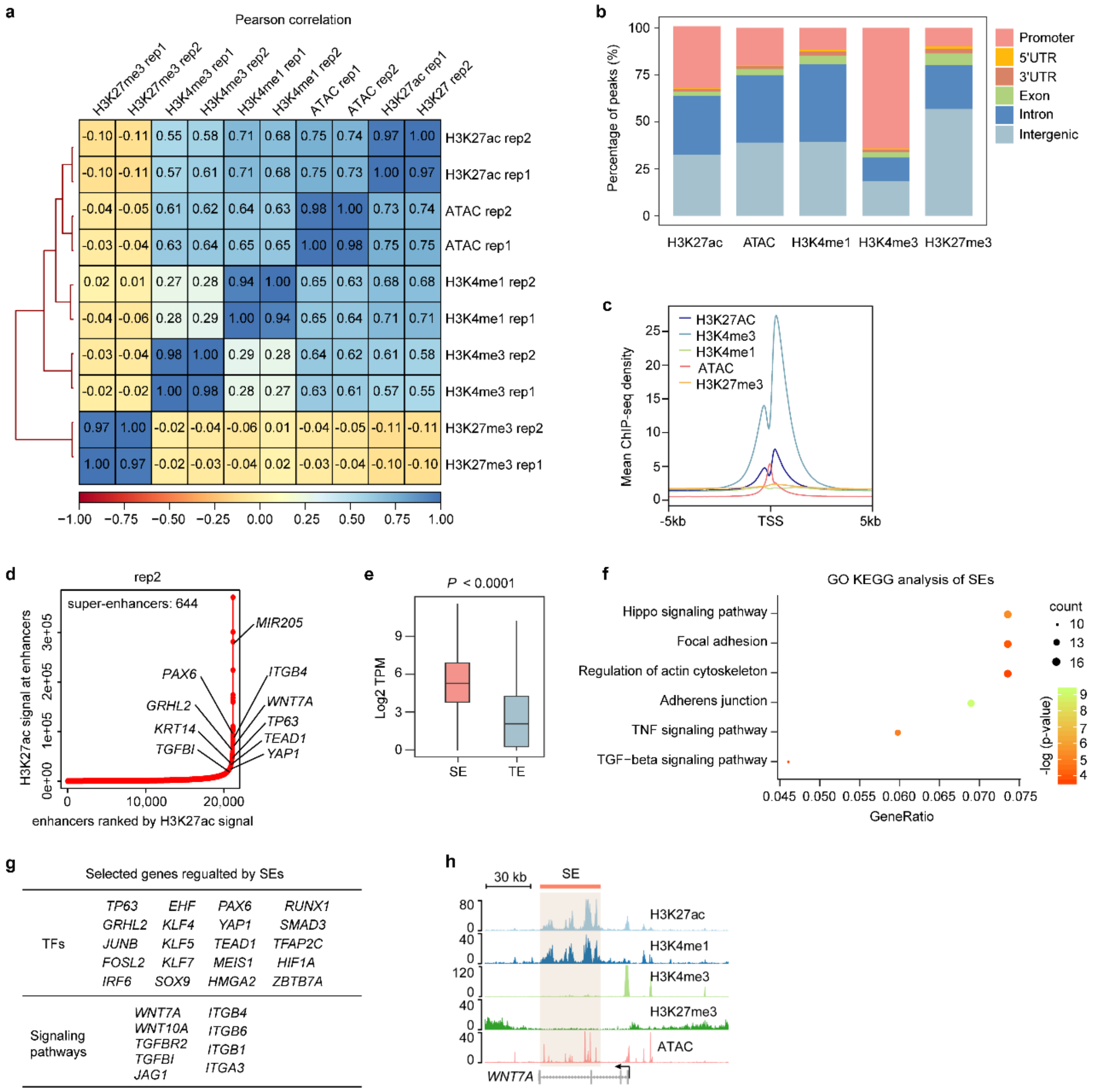

Supplementary Fig. 1 Characteristic of LSC-specific SEs

a Clustered heatmap of pearson correlation for the indicated ChIP-seq and ATAC-seq data based on the normalized read coverages across the genome-wide regions. **b** Barplots showing the distribution patterns of ATAC, H3K27ac, H3K4me1, H3K4me3 and H3K27me3 throughout the genome. **c** Metaplots of average ATAC-seq and the indicated ChIP-seq density at the transcription start sites (TSSs). **d** Ranked enhancer plots defined by H3K27ac in the repetition (rep) 2 LSCs. Enhancers above the inflection point of the curve have exceptionally strong H3K27ac signals and are defined as SEs. The selected genes are SE-associated genes. **e** Boxplots showing the global expression levels of SE- and TE-associated genes ($P < 0.0001$). SE: n = 520

genes; TE: n = 20795 genes. TPM, transcripts per kilobase million. *P* value was calculated using an unpaired two-tailed t test. Bar represents median, box indicates 25% and 75%, and whiskers indicate minimum and maximum values. **f** GO: Kyoto Encyclopedia of Genes and Genomes (KEGG) pathway enrichment analysis of SE genes. **g** List of the selected SE-regulated genes. **h** Genome browser tracks for the indicated ChIP-seq and ATAC-seq signals across the *WNT7A* locus in LSCs.

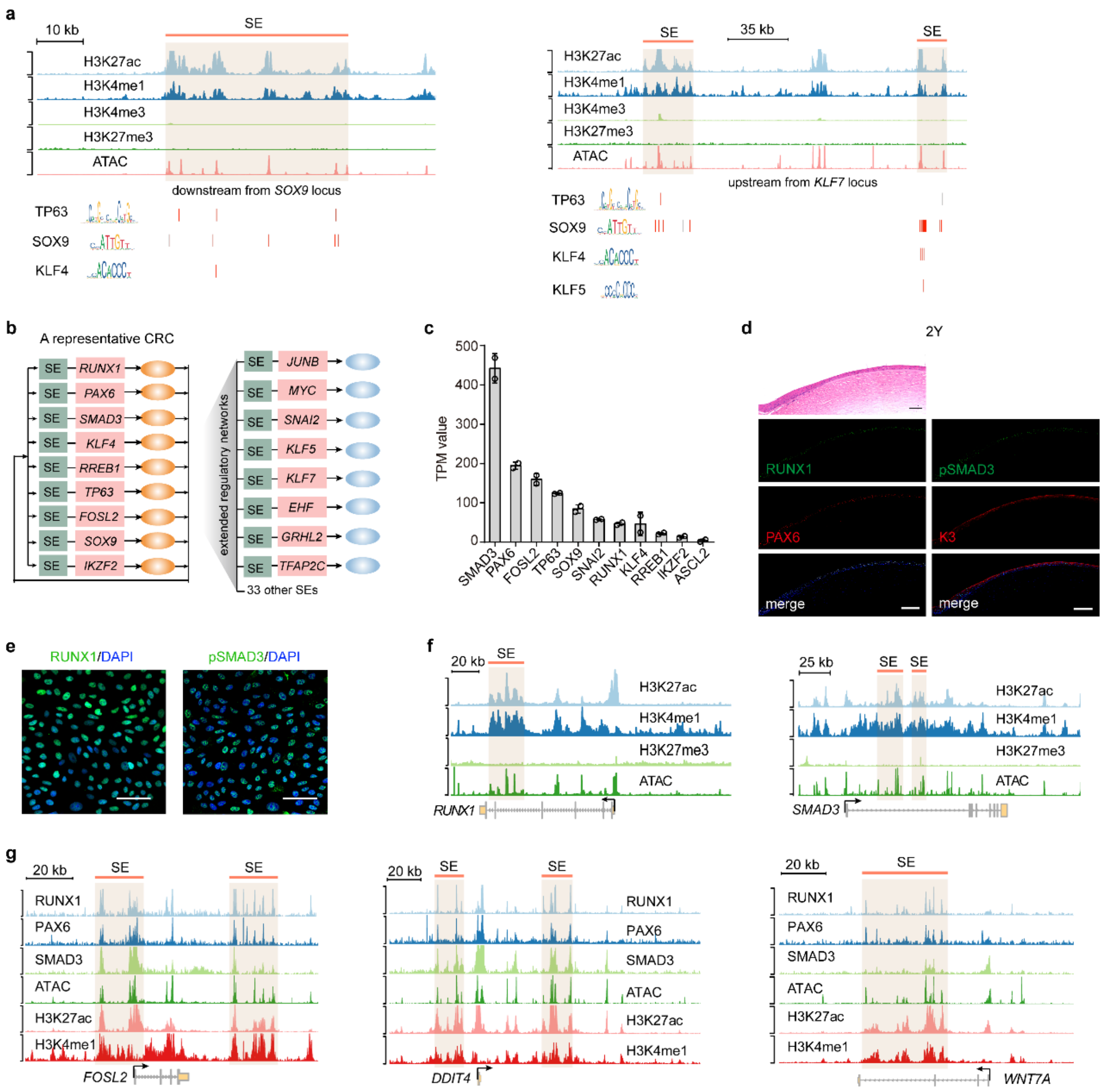

Supplementary Fig. 2 The RPS-established CRC

a Genome browser tracks for the indicated ChIP-seq and ATAC-seq signals at the indicated SE loci with logos and positions of the enriched TF motifs shown. **b** A representative CRC in LSCs. These CRC TFs form an interconnected autoregulatory loop and co-regulate an extended SE network. **c** TPM values of the selected CRC TFs. Values are shown as means ± SD (n = 2 biologically independent samples). **d** H&E and immunofluorescence staining of the indicated genes in 2-year-old normal cornea and limbus. Scale bars, 200 μm. **e** Immunofluorescence staining of RUNX1 and pSMAD3 in cultured primary LSCs. Scale bars, 100 μm. **f** Genome browser tracks for the indicated ChIP-seq and ATAC-seq signals across the *RUNX1* and *SMAD3* loci. **g** Genome browser tracks for the indicated ChIP-seq and ATAC-seq signals

across the indicated SE loci in LSCs.

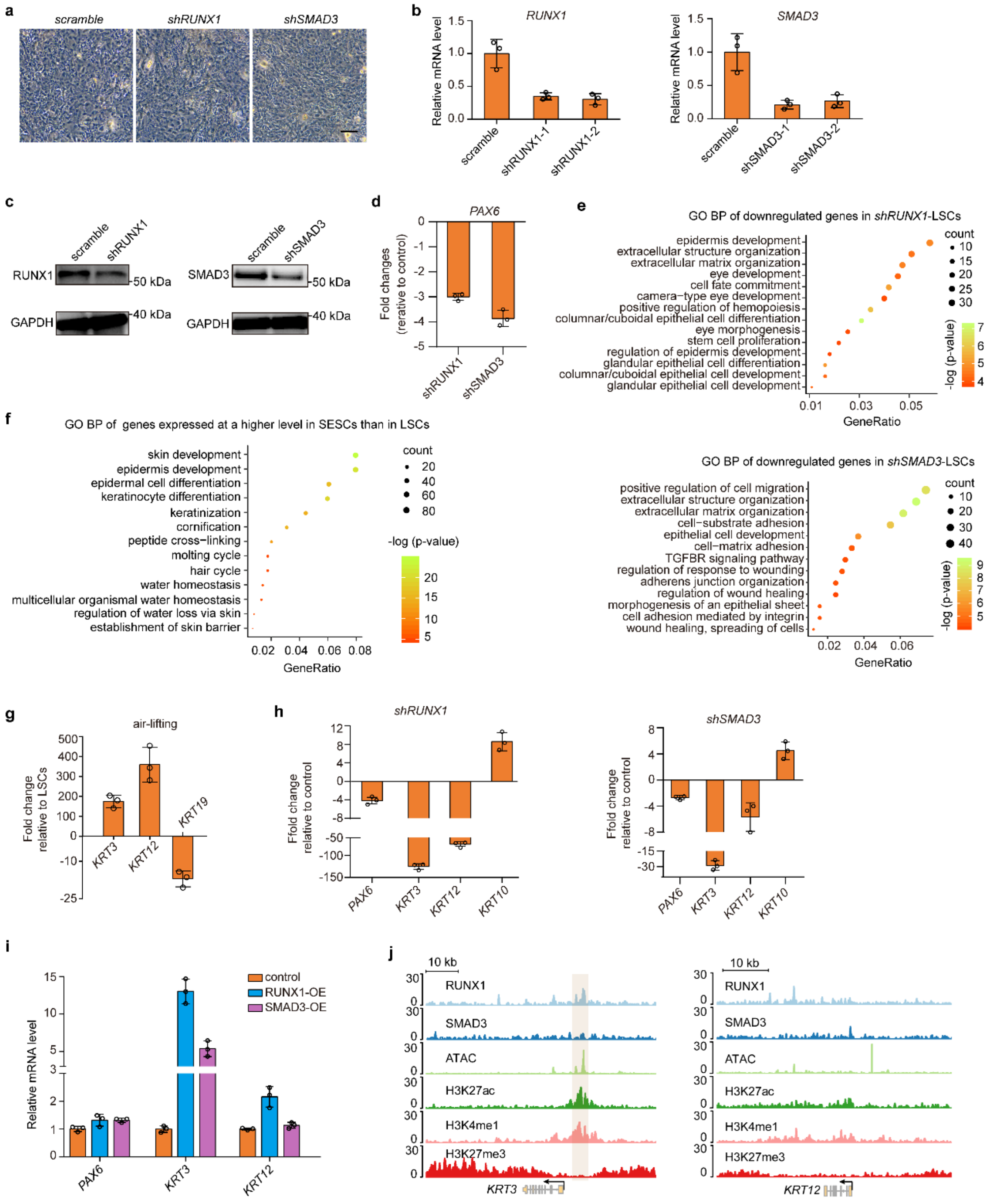

Supplementary Fig. 3 RUNX1 and SMAD3 determine the corneal epithelial identity

a Phase-contrast images of LSCs treated with scrambled shRNA, *shRUNX1*, and *shSMAD3*. Scale bar, 200 μm. **b** Real-time quantitative PCR (qRT-PCR) analysis of the KD efficiency of *RUNX1* and *SMAD3*. Values are shown as means ± SD (n = 3 biologically independent experiments). **c** Western blot analysis of RUNX1 and SMAD3 in LSCs treated with the indicated shRNAs. **d** qRT-PCR analysis of *PAX6* expression after *RUNX1* or *SMAD3* KD in LSCs;

Values are shown as means ± SD (n = 3 biologically independent experiments). **e** GO BP analysis of the downregulated genes in *RUNX1*-depleted and *SMAD3*-depleted LSCs (pvalueCutoff = 0.01 and qvalueCutoff = 0.05). **f** GO BP analysis for the genes expressed at a higher level in SESCs than in LSCs (pvalueCutoff = 0.01 and qvalueCutoff = 0.05). **g** qRT-PCR analysis of the relative expression levels of *KRT3*, *KRT12*, and *KRT19* in air-lifting-induced cornea epithelium sheet compared to those in LSCs. Values are shown as means ± SD (n = 3 biologically independent experiments). **h** qRT-PCR analysis for fold-changes in the expression of the indicated genes in the differentiated corneal epithelium sheets treated with *shRUNX1* or *shSMAD3* versus scrambled shRNA; Values are shown as means ± SD (n = 3 biologically independent experiments). **i** qRT-PCR analysis of the relative expression levels of *PAX6*, *KRT3*, and *KRT12* in RUNX1-overexpressed (RUNX1-OE) and SMAD3-overexpressed (SMAD3-OE) LSCs. Values are shown as means ± SD (n = 3 biologically independent experiments). **j** Genome browser tracks for the indicated ChIP-seq and ATAC-seq signals across the *KRT3* and *KRT12* loci.

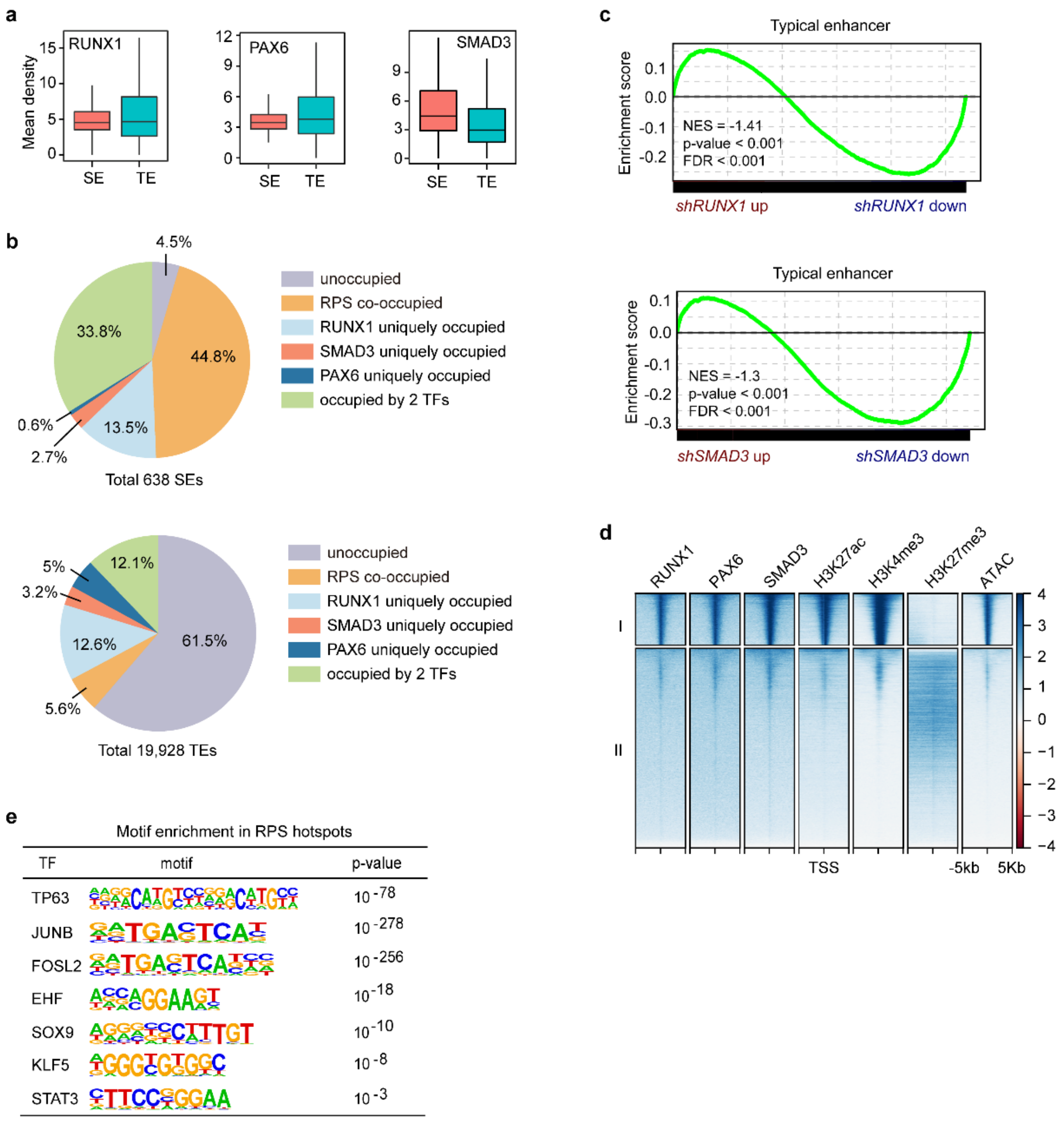

Supplementary Fig. 4 RPS co-occupy *cis*-regulatory elements

a Boxplots of average ChIP-seq signals for RPS at TEs and SEs in LSCs. *P* values for RUNX1 ($P = 0.8063$), PAX6 ($P = 0.0031$), and SMAD3 ($P < 0.0001$) were calculated using unpaired two-tailed t tests. Bar represents median, box indicates 25% and 75%, and whiskers indicate minimum and maximum values. **b** Pie charts showing the percentages of SEs and TEs occupied by RPS in LSCs. **c** GSEA of TE-associated gene set in scrambled shRNA- versus *shRUNX1*-treated LSCs and scrambled shRNA- versus *shSMAD3*-treated LSCs. NES, normalized enrichment score. **d** Heatmaps grouped into two clusters by k-means algorithm for the indicated ChIP-seq and ATAC-seq signals at the TSSs in LSCs. **e** Motif enrichment at the center of RPS hotspots.

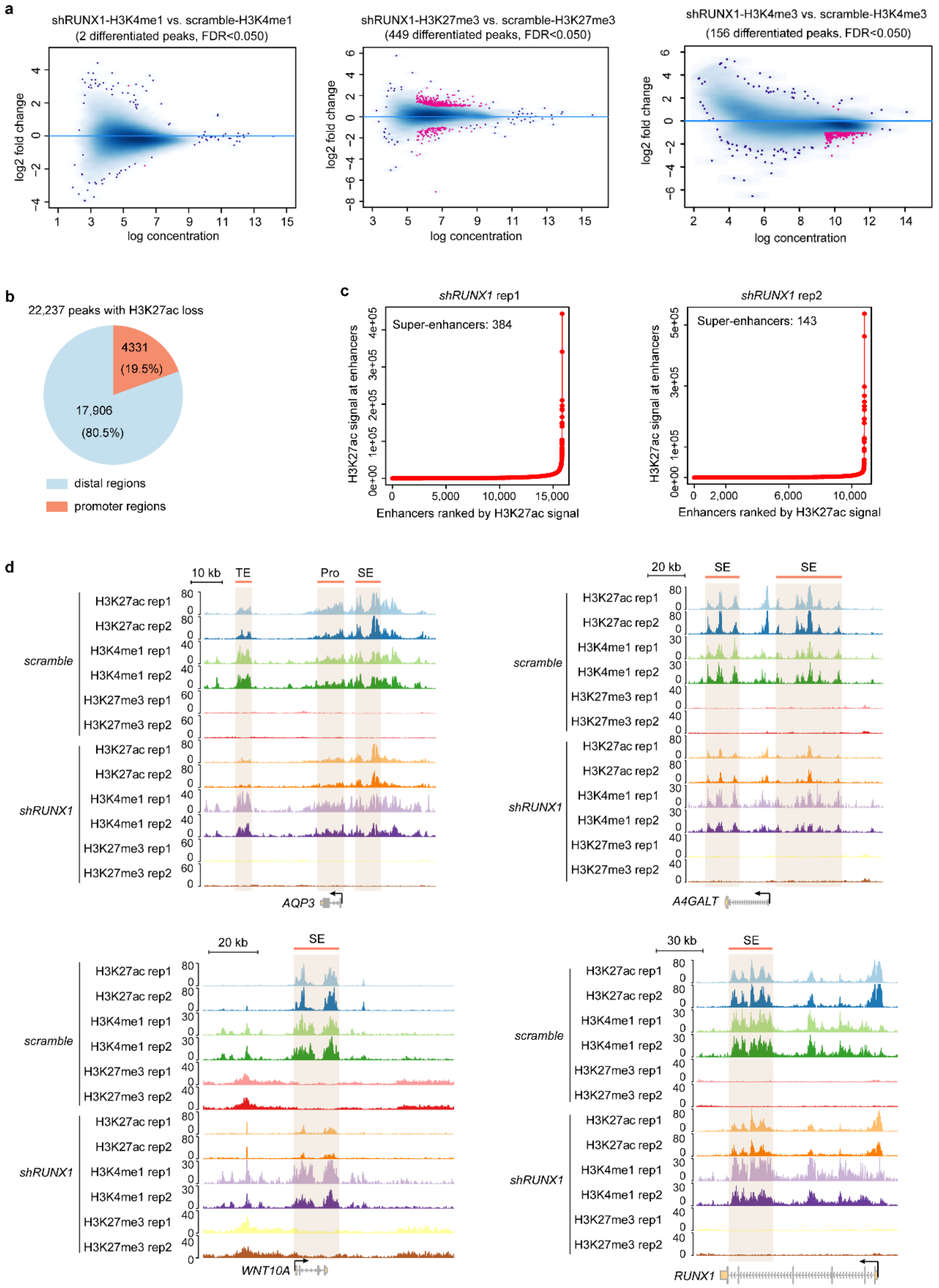

Supplementary Fig. 5 Loss of *RUNX1* induces conversion of SEs into TEs

a Scatterplots of H3K4me1, H3K27me3, and H3K4me3 peaks in *shRUNX1*-versus scrambled shRNA-treated LSCs. Sites identified as significantly differentially bound (fold change ≥ 2, FDR < 0.05) are shown in red. **b** Pie chart showing the percentages of decreased H3K27ac peaks induced by *RUNX1*

loss in promoter and distal regions. **c** Ranked enhancer plots defined by H3K27ac in *shRUNX1*-treated LSCs. Enhancers above the inflection point of the curve have exceptionally strong H3K27ac signals and are defined as SEs. **d** Genome browser tracks for the indicated ChIP-seq signals across the *AQP3*, *A4GALT*, *WNT10A,* and *RUNX1* loci in scrambled shRNA- and *shRUNX1*-treated LSCs.

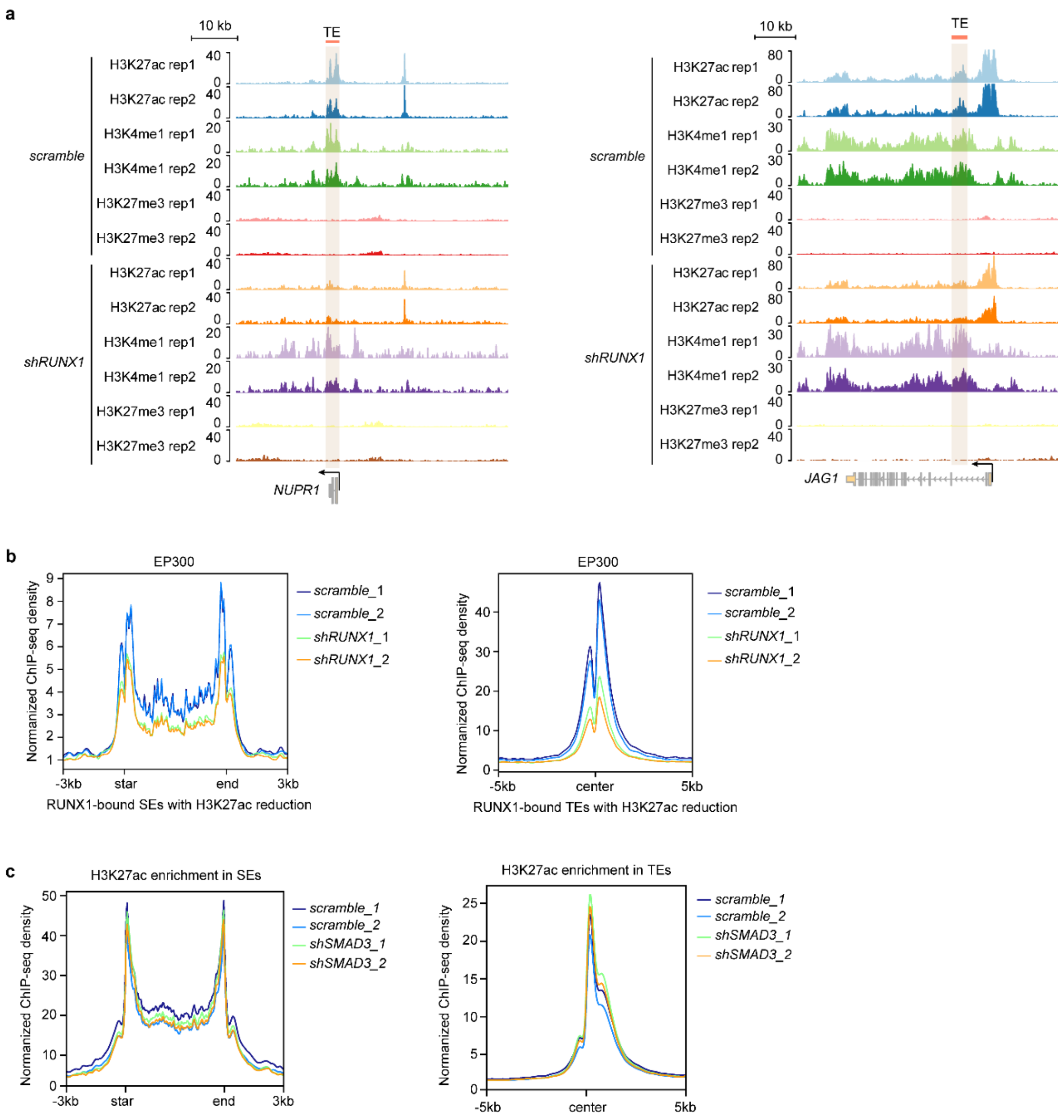

Supplementary Fig. 6 Loss of *RUNX1* diminishes H3K27ac level at TEs and promoters

a Genome browser tracks for the indicated ChIP-seq signals across the *NUPR1,* and *JAG1* loci in scrambled shRNA- and *shRUNX1*-treated LSCs. **b** Metaplots of average EP300 density across the RUNX1-bound SEs and TEs with reduced H3K27ac levels (induced by *RUNX1* depletion) in *shRUNX1*- and scrambled shRNA-treated LSCs. **c** Metaplots of average H3K27ac density across the SEs and TEs in *shSMAD3*- and scrambled shRNA-treated LSCs.

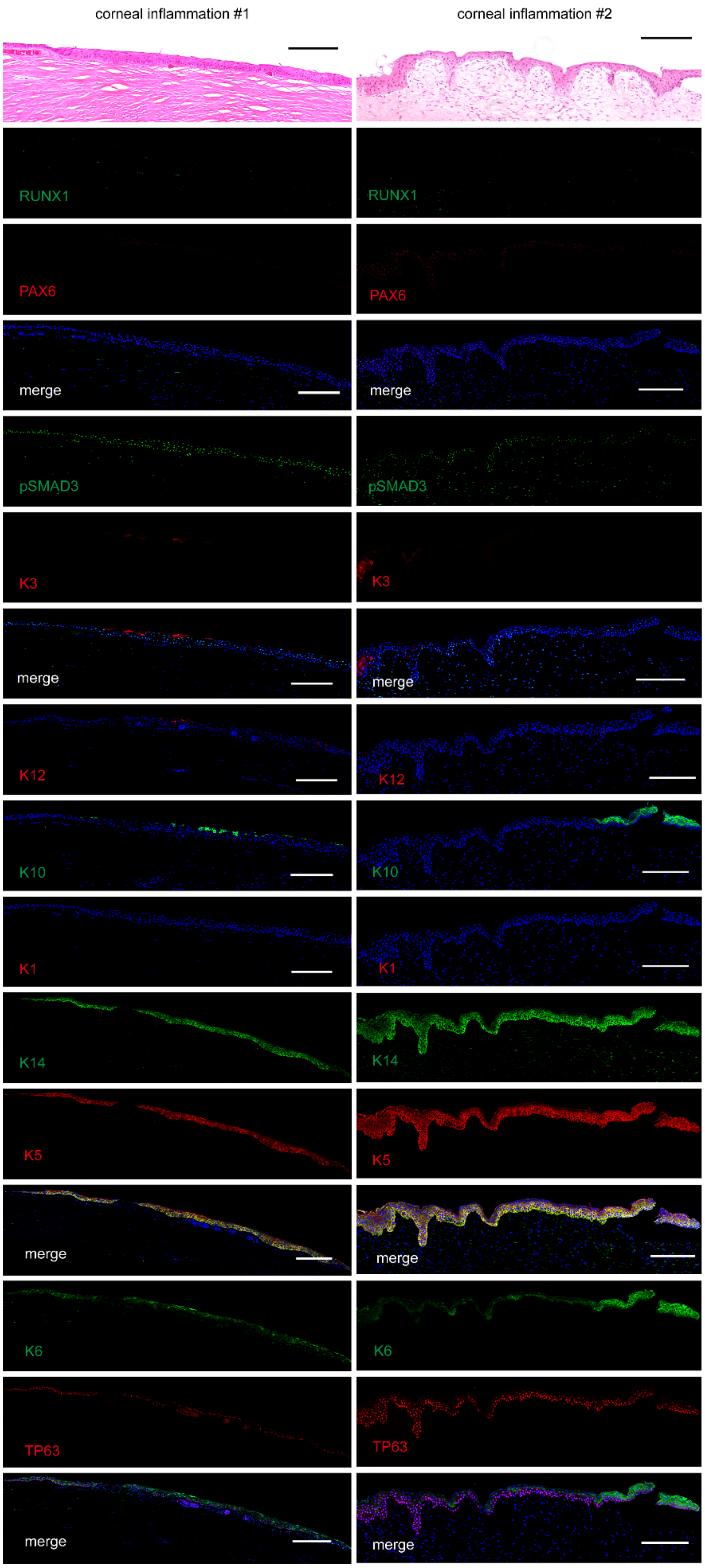

Supplementary Fig. 7 Pathological changes in corneal inflammatory tissues

H&E staining and immunofluorescence analysis of the indicated genes in corneal inflammatory tissues. Scale bars, 200 μm. Patient #1: 27 years old, male. Patient #2: 43 years old, male.

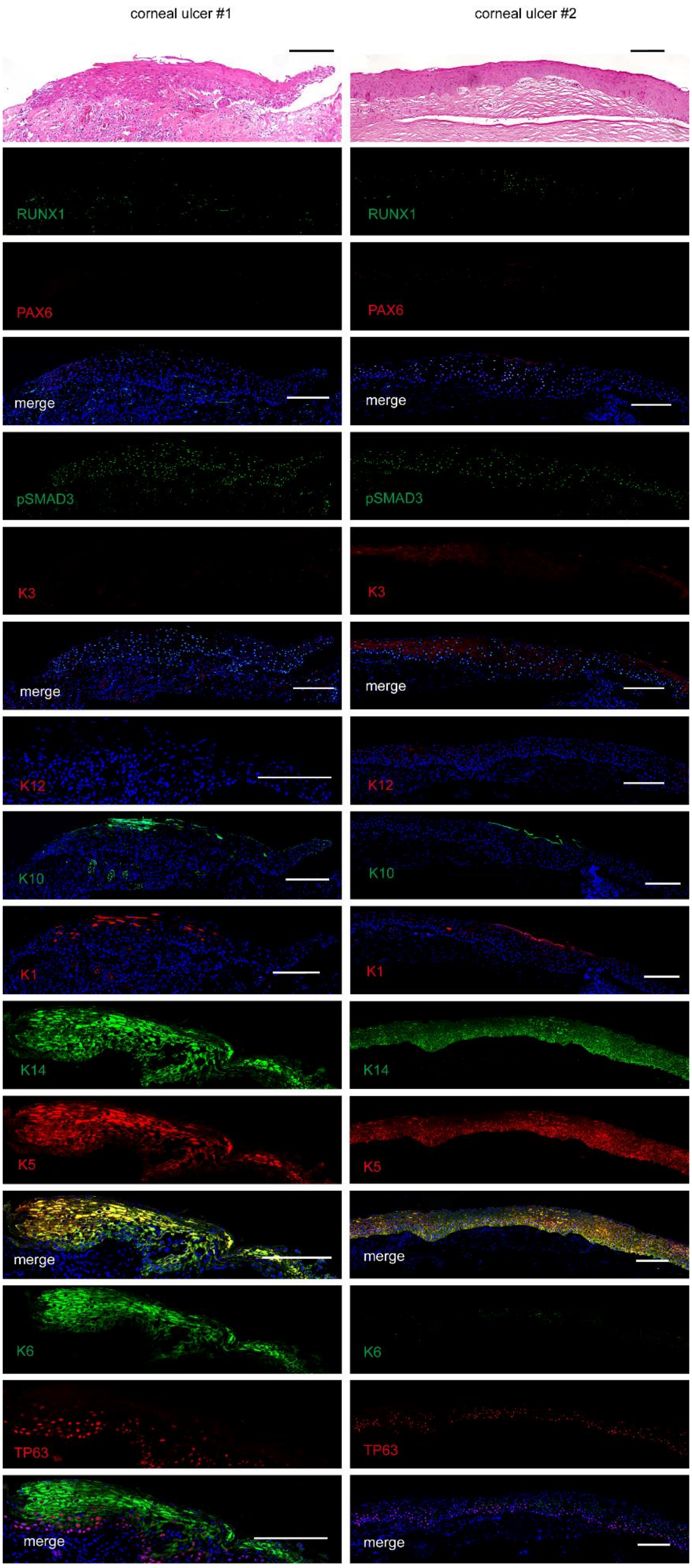

Supplementary Fig. 8 Pathological changes in corneal ulcer tissues

H&E staining and immunofluorescence analysis of the indicated genes in corneal ulcer tissues. Scale bars, 200 μm. Patient #1: 64 years old, male. Patient #2: 58 years old, male.

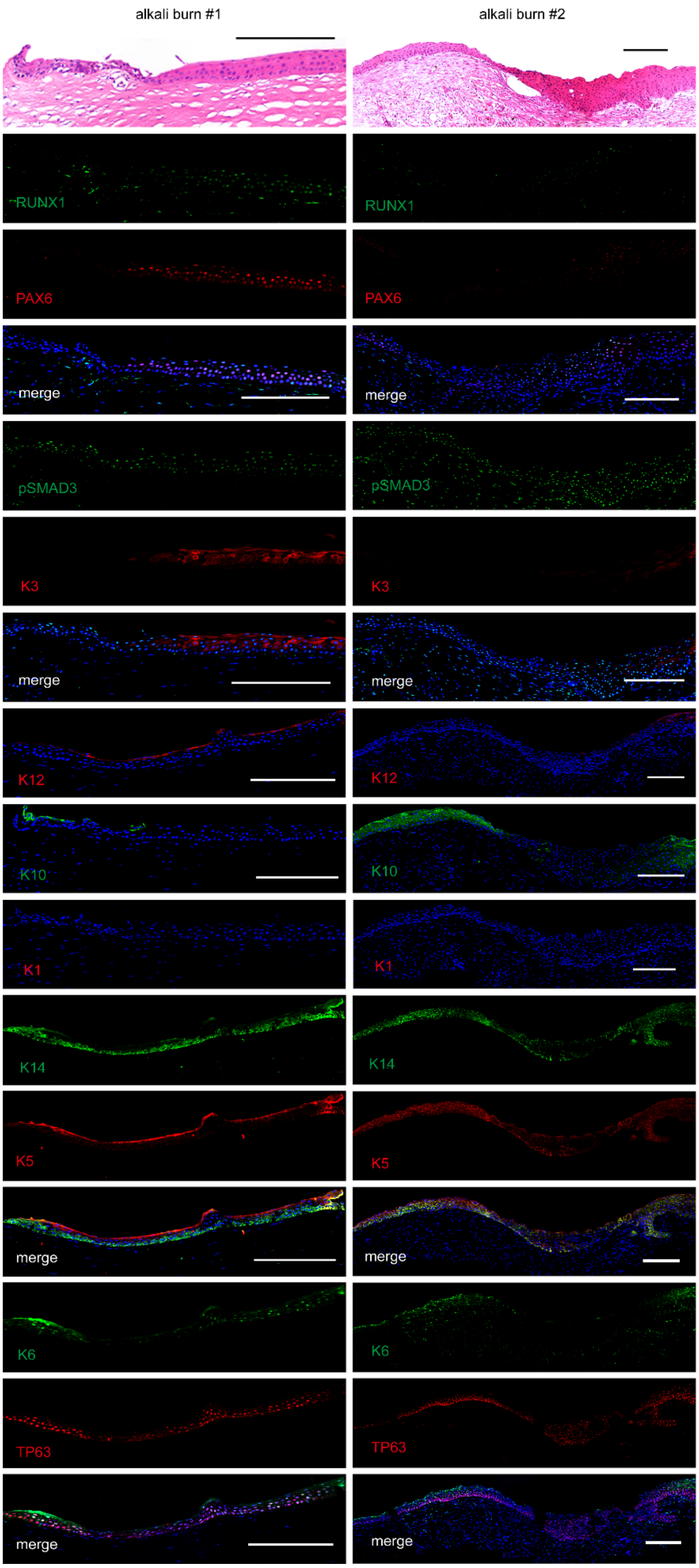

Supplementary Fig. 9 Pathological changes in corneal alkali burn tissues

H&E staining and immunofluorescence analysis of the indicated genes in corneal alkali burn tissues. Scale bars, 200 μm. Patient #1: 7 years old, female. Patient #2: 68 years old, female.

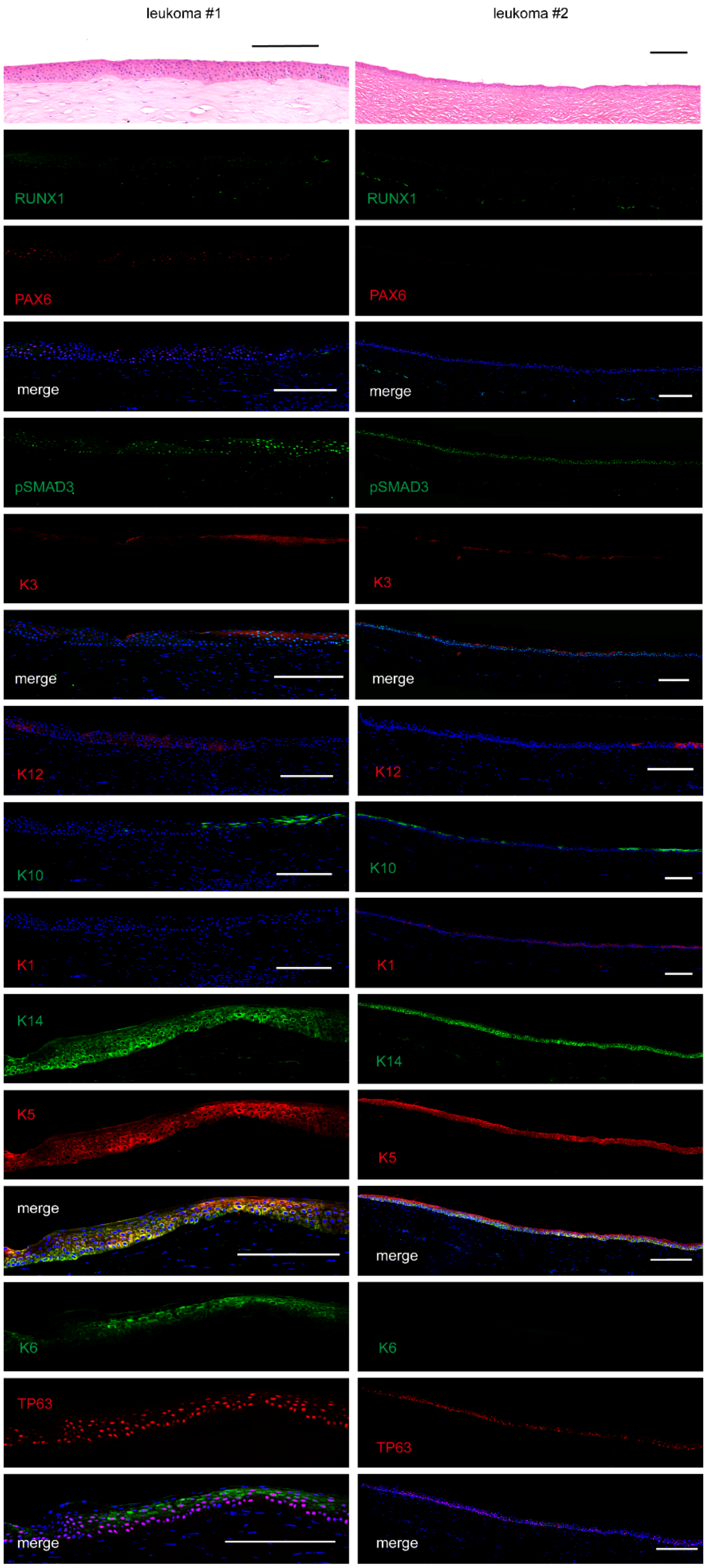

Supplementary Fig. 10 Pathological changes in leukoma tissues

H&E staining and immunofluorescence analysis of the indicated genes in leukoma tissues. Scale bars, 200 μm. Patient #1: 13 years old, male. Patient #2: 39 years old, male.

a

Patient information and gene expression

	inflammation			ulcer			alkali burn			leukoma		
patient	#1	#2	#3	#1	#2	#3	#1	#2	#3	#1	#2	#3
sex	female	male	male	female	male	male	male	female	female	female	male	male
age	47	27	43	62	64	58	33	7	68	51	13	39
RUNX1	-/+	-	-	-	-	-/+	-	-/+	-/+	-	-	-
PAX6	-/+	-	-/+	-/+	-	-/+	-	-/+	-/+	-	-/+	-
SMAD3	+	+	+	+	+	+	+	+	+	+	+	+
KRT3	-/+	-	-	-/+	-	-/+	-	-/+	-	-/+	-/+	-/+
KRT12	-/+	-	-	-/+	-	-	-	-/+	-	-/+	-/+	-/+
KRT1	-	-	-	-	-/+	-/+	-/+	-	-	-	-	-/+
KRT10	-/+	-/+	-/+	-/+	-/+	-/+	-/+	-/+	-/+	-/+	-/+	-/+
KRT14	+	+	+	+	+	+	+	+	+	+	+	+
KRT5	+	+	+	+	+	+	+	+	+	+	+	+
KRT6	-/+	+	+	+	+	-/+	+	+	-/+	+	+	-
TP63	+	+	+	+	+	+	+	+	+	+	+	+

-/+ Negative in partial area

+ Positive

- Negative

b normal cornea

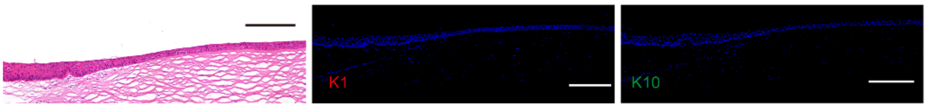

c epidermis

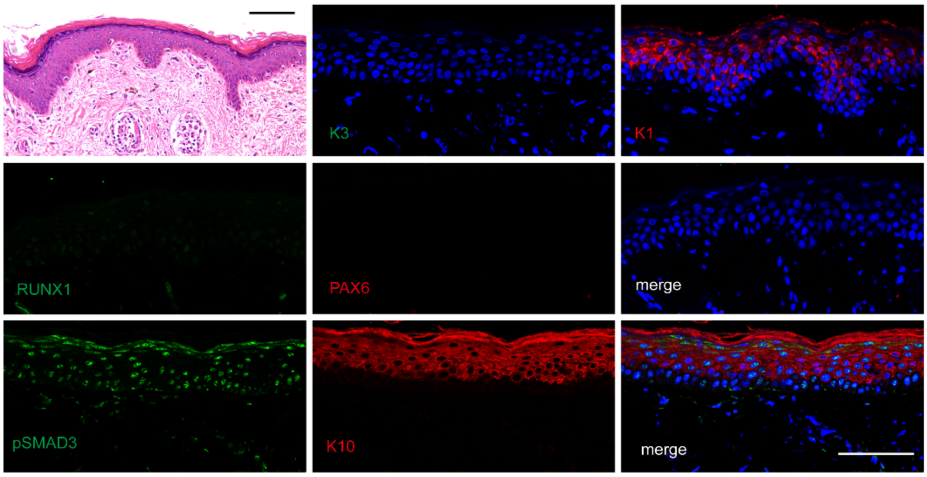

Supplementary Fig. 11 Gene expression in human corneal diseases, normal corneal epithelium, and epidermis. a Patient information and gene expression of the examined corneal diseased tissues. **b** H&E staining and immunofluorescence analysis of KRT1 and KRT10 in normal adult corneal epithelium. Scale bars, 200 μm. **c** H&E staining and immunofluorescence analysis of the indicated genes in adult epidermis. Scale bars, 100 μm.

Supplementary Table 1 Human qRT-PCR primers

Primer name	Sequence: 5’-3’
PAX6-F	CAATTCCACAACCCACCACAC
PAX6-R	CGCTGTAGGTGTTTGTGAGGG
GAPDH-F	CGGAGTCAACGGATTTGGTC
GAPDH-R	CCTGGAAGATGGTGATGGGAT
KRT12-F	AGCAGAATCGGAAGGACGCTGA
KRT12-R	ACCTCGCTCTTGCTGGACTGAA
KRT3-F	CCTCTACGACGCTGAGCTATC
KRT3-R	CCAGGGAGCGATTATTGTCCAT
KRT10-F	TCCTACTTGGACAAAGTTCGGG
KRT10-R	CCCCTGATGTGAGTTGCCA
KRT1-F	CAGCATCATTGCTGAGGTCAAGG
KRT1-R	CATGTCTGCCAGCAGTGATCTG
SMAD3-F	GCGTGCGGCTCTACTACATC
SMAD3-R	GCACATTCGGGTCAACTGGTA
RUNX1-F	TCTTCACAAACCCACCGCAA
RUNX1-R	CTGCCGATGTCTTCGAGGTTC
KRT19-F	AGCTAGAGGTGAAGATCCGCGA
KRT19-R	GCAGGACAATCCTGGAGTTCTC
