# Supplementary material for: Core transcription regulatory circuitry orchestrates corneal epithelial homeostasis
Source: Nat Commun. 2021 Jan 18;12:420. doi: 10.1038/s41467-020-20713-z (PMC7814021; doi:10.1038/s41467-020-20713-z)
Supplement: Supplementary file 2 — Reporting Summary [file 41467_2020_20713_MOESM2_ESM.pdf]

nature research

Corresponding author(s): Hong Ouyang

Last updated by author(s): Dec 8, 2020

Reporting Summary

Nature Research wishes to improve the reproducibility of the work that we publish. This form provides structure for consistency and transparency in reporting. For further information on Nature Research policies, see our Editorial Policies and the Editorial Policy Checklist.

Statistics

For all statistical analyses, confirm that the following items are present in the figure legend, table legend, main text, or Methods section.

n/a	Confirmed	
☐	☒	The exact sample size (*n*) for each experimental group/condition, given as a discrete number and unit of measurement
☐	☒	A statement on whether measurements were taken from distinct samples or whether the same sample was measured repeatedly
☐	☒	The statistical test(s) used AND whether they are one- or two-sided<br>*Only common tests should be described solely by name; describe more complex techniques in the Methods section.*
☐	☒	A description of all covariates tested
☐	☒	A description of any assumptions or corrections, such as tests of normality and adjustment for multiple comparisons
☐	☒	A full description of the statistical parameters including central tendency (e.g. means) or other basic estimates (e.g. regression coefficient) AND variation (e.g. standard deviation) or associated estimates of uncertainty (e.g. confidence intervals)
☐	☒	For null hypothesis testing, the test statistic (e.g. *F*, *t*, *r*) with confidence intervals, effect sizes, degrees of freedom and *P* value noted<br>*Give P values as exact values whenever suitable.*
☒	☐	For Bayesian analysis, information on the choice of priors and Markov chain Monte Carlo settings
☐	☒	For hierarchical and complex designs, identification of the appropriate level for tests and full reporting of outcomes
☐	☒	Estimates of effect sizes (e.g. Cohen's *d*, Pearson's *r*), indicating how they were calculated

Our web collection on statistics for biologists contains articles on many of the points above.

Software and code

Policy information about availability of computer code

Data collection	trimmomatic tool (version 0.36), BWA (version 0.7.17), MACS2 (version 2.1.1), DiffBind package (version 2.8.0), STAR software (version 2.6.1a), DESeq2 (version 1.20.0), HOMER (v4.9.1), deepTools (v3.0.2), clusterprofiler (v3.8.1), ZEISS LSM 800 Confocal Laser Scanning Microscope
Data analysis	Data representation and statistical analysis: Graphpad Prism 7 and Microsoft Office Excel 2017. All analysis processes for Next-generation sequencing data are stated in the method section.

For manuscripts utilizing custom algorithms or software that are central to the research but not yet described in published literature, software must be made available to editors and reviewers. We strongly encourage code deposition in a community repository (e.g. GitHub). See the Nature Research guidelines for submitting code & software for further information.

Data

Policy information about availability of data

All manuscripts must include a data availability statement. This statement should provide the following information, where applicable:

- Accession codes, unique identifiers, or web links for publicly available datasets
- A list of figures that have associated raw data
- A description of any restrictions on data availability

All data supporting the findings of this study are available within the paper and its supplementary information file. Source data are provided with this paper. All sequencing data are available through Gene Expression Omnibus accession number: GSE156273.

Field-specific reporting

Please select the one below that is the best fit for your research. If you are not sure, read the appropriate sections before making your selection.

- [x] Life sciences
- [ ] Behavioural & social sciences
- [ ] Ecological, evolutionary & environmental sciences

For a reference copy of the document with all sections, see nature.com/documents/nr-reporting-summary-flat.pdf

Life sciences study design

All studies must disclose on these points even when the disclosure is negative.

Sample size	Cellular and molecular biology experiments were performed in triplicate and the next-generation sequencing experiments (ChIP-seq, RNA-seq and ATAC-seq) were performed in two replicates, which is sufficient to determine reproducible results based on extensive experience and is widely accepted.
Data exclusions	No data were excluded from the analyses.
Replication	All results were reliably reproduced in multiple independent experiments as indicated in the figure legends.
Randomization	All tissues were selected randomly.
Blinding	All experiments were objectives and did not require blinding analysis

Reporting for specific materials, systems and methods

We require information from authors about some types of materials, experimental systems and methods used in many studies. Here, indicate whether each material, system or method listed is relevant to your study. If you are not sure if a list item applies to your research, read the appropriate section before selecting a response.

Materials & experimental systems

n/a	Involved in the study	
[ ]	[x]	Antibodies
[x]	[ ]	Eukaryotic cell lines
[x]	[ ]	Palaeontology and archaeology
[x]	[ ]	Animals and other organisms
[ ]	[x]	Human research participants
[x]	[ ]	Clinical data
[x]	[ ]	Dual use research of concern

Methods

n/a	Involved in the study	
[ ]	[x]	ChIP-seq
[x]	[ ]	Flow cytometry
[x]	[ ]	MRI-based neuroimaging

Antibodies

Antibodies used	Antibodies used for immunofluorescence: anti-PAX6 (BioLegend, 901301,1:500), anti-PAX6 (Sigma, AMAB91372,1:500), anti-RUNX1 (Abcam, ab23980,1:500), anti-pSMAD3 (Abcam, ab52903,1:500), anti-K19 (Thermo Scientific, MS-1902-P,1:1000), anti-p63 (BioLegend, 619002,1:500), anti-Ki67 (CST, 9129S,1:500), anti-Cytokeratin 1 (Invitrogen, MA1-06312,1:1000), anti-Cytokeratin 10 (Invitrogen, MA1-06319,1:1000), anti-Cytokeratin 3 (Abcam, ab68260,1:1000), anti-Keratin 12 (Abcam, ab124975,1:1000), anti-rabbit IgG (Alexa Fluor 488 Conjugate, CST, 4412S,1:1000), anti-mouse IgG (Alexa Fluor 488 Conjugate, CST, 4408S,1:1000), anti-rabbit IgG (Alexa Fluor 594 Conjugate, CST, 8889S,1:1000), and anti-mouse IgG (Alexa Fluor 594 Conjugate, CST, 8890S,1:1000).<br>Antibodies used for immunoprecipitation (10μg/IP): anti-PAX6 (IP, BioLegend, 901301), anti-PAX6 (WB, Invitrogen, PA1-801,1:1000), anti-RUNX1 (IP, Abcam, ab23980), anti-RUNX1 (WB, BioLegend, 659302,1:1000), anti-SMAD3 (WB, Invitrogen, MA5-15663,1:1000), and anti-pSMAD3 (IP, Abcam, ab52903).<br>Antibodies used for ChIP-seq (5μg/ChIP): anti-H3K27ac (Millipore, 07-360), anti-H3K27me3 (CST, 9733s), anti-H3K4me1 (Active Motif, 39297), anti-H3K4me3 (CST, 9751S), anti-PAX6 (BioLegend, 901301), anti-RUNX1 (Abcam, ab23980), anti-SMAD3 (Abcam, ab28379), anti-EP300 (Abcam, ab14984).
Validation	Antibodies used in our study were validated as noted by suppliers. Antibody validation information can be found on manufacturers' website and in previous publications.

Human research participants

Policy information about studies involving human research participants

Population characteristics	Supplementary Fig.11a lists the age and gender of each patient donor of diseased corneal tissues. Normal human skin biopsies (30-40 yeas old) and limbus (30-50 years old) from donors were used.
Recruitment	This study did not involve new recruitment of patients. Diseased corneal tissues were obtained from keratoplasty patients. Normal skin biopsies were obtained from eye lids of donors. Normal human limbus of donors were obtained from eye bank of Zhongshan Ophthalmic Center.
Ethics oversight	All human tissues were obtained with the approval of the Ethics Committee of Zhongshan Ophthalmic Center of Sun Yat-sen University.

Note that full information on the approval of the study protocol must also be provided in the manuscript.

ChIP-seq

Data deposition

☒ Confirm that both raw and final processed data have been deposited in a public database such as GEO.

☒ Confirm that you have deposited or provided access to graph files (e.g. BED files) for the called peaks.

Data access links<br>*May remain private before publication.*	https://www.ncbi.nlm.nih.gov/geo/query/acc.cgi?acc=GSE156273
Files in database submission	H3K27ac_rep1<br>H3K27ac_rep2<br>H3K4me1_rep1<br>H3K4me1_rep2<br>H3K4me3_rep1<br>H3K4me3_rep2<br>H3K27me3_rep1<br>H3K27me3_rep2<br>scr_H3K27ac_rep1<br>scr_H3K27ac_rep2<br>shRUNX1_H3K27ac_rep1<br>shRUNX1_H3K27ac_rep2<br>scr_H3K4me1_rep1<br>scr_H3K4me1_rep2<br>shRUNX1_H3K4me1_rep1<br>shRUNX1_H3K4me1_rep2<br>scr_H3K4me3_rep1<br>scr_H3K4me3_rep2<br>shRUNX1_H3K4me3_rep1<br>shRUNX1_H3K4me3_rep2<br>scr_H3K27me3_rep1<br>scr_H3K27me3_rep2<br>shRUNX1_H3K27me3_rep1<br>shRUNX1_H3K27me3_rep2<br>RUNX1_rep1<br>RUNX1_rep2<br>PAX6_rep1<br>PAX6_rep2<br>SMAD3_rep1<br>SMAD3_rep2
Genome browser session<br>(e.g. UCSC)	GSE156273

Methodology

Replicates	Two independent experimental replications were performed.
Sequencing depth	6Gb paired-end data were generated.
Antibodies	anti-H3K27ac (Millipore, 07-360), anti-H3K27me3 (CST, 9733s), anti-H3K4me1 (Active Motif, 39297), anti-H3K4me3 (CST, 9751S), anti-PAX6 (BioLegend, 901301), anti-RUNX1 (Abcam, ab23980), and anti-SMAD3 (Abcam, ab28379)
Peak calling parameters	-f BAMPE -B --SPMR -q 0.001 --call-summits --fix-bimodal --seed 11521 --extsize 200

Data quality	FastQC(v0.11.8) was used to assess the data quality
Software	trimmomatic tool (version 0.36), BWA (version 0.7.17), MACS2 (version 2.1.1), DiffBind package (version 2.8.0), deepTools (v3.0.2)
